# Panarthropod *tiptop/teashirt* and *spalt* orthologs and their potential role as “trunk”-selector genes

**DOI:** 10.1186/s13227-021-00177-y

**Published:** 2021-06-02

**Authors:** Brenda I. Medina-Jiménez, Graham E. Budd, Ralf Janssen

**Affiliations:** Department of Earth Sciences, Palaeobiology, Uppsala University, Villavägen 16, Uppsala, Sweden

**Keywords:** Arthropod development, Hox, Homeotic gene, Trunk-selector, Panarthropoda, Onychophora

## Abstract

**Background:**

In the vinegar fly *Drosophila melanogaster*, the homeodomain containing transcription factor Teashirt (Tsh) appears to specify trunk identity in concert with the function of the Hox genes. While in *Drosophila* there is a second gene closely related to *tsh*, called *tiptop* (*tio*), in other arthropods species only one copy exists (called *tio/tsh*). The expression of *tsh* and *tio/tsh*, respectively, is surprisingly similar among arthropods suggesting that its function as trunk selector gene may be conserved. Other research, for example on the beetle *Tribolium castaneum*, questions even conservation of Tsh function among insects. The zinc-finger transcription factor Spalt (Sal) is involved in the regulation of *Drosophila tsh*, but this regulatory interaction does not appear to be conserved in *Tribolium* either. Whether the function and interaction of *tsh* and *sal* as potential trunk-specifiers, however, is conserved is still unclear because comparative studies on *sal* expression (except for *Tribolium*) are lacking, and functional data are (if at all existing) restricted to Insecta.

**Results:**

Here, we provide additional data on arthropod *tsh* expression, show the first data on onychophoran *tio/tsh* expression, and provide a comprehensive investigation on *sal* expression patterns in arthropods and an onychophoran.

**Conclusions:**

Our data support the idea that *tio/tsh* genes are involved in the development of “trunk” segments by regulating limb development. Our data suggest further that the function of Sal is indeed unlikely to be conserved in trunk vs head development like in *Drosophila*, but early expression of *sal* is in line with a potential homeotic function, at least in Arthropoda.

**Supplementary Information:**

The online version contains supplementary material available at 10.1186/s13227-021-00177-y.

## Introduction

Segmentation and tagmosis, the subdivision of the anterior–posterior (AP) body axis into serially homologous units (segments) and functional body units (tagmata), represent key innovations in the evolution of arthropods, that have enabled them to become the most successful group of animals [8]. The subdivision of the AP axis allowed evolutionary flexibility that led to the adaptation to almost all ecological niches on the planet, “simply” by altering a segment’s morphology without having to disturb the animal’s overall body plan.

In the vinegar fly *Drosophila melanogaster*, the specific identity of each segment is under control of the Hox genes, a group of closely related homeodomain containing transcription factors (e.g. [9, 39, 42]). Ectopic expression of Hox genes and loss of Hox gene function has proven to result in homeotic transformations, the change of a segment from one fate into that of another segment (e.g. [28, 59]). Subsequent work in representatives of other groups of arthropods such as other hexapods, crustaceans, myriapods and chelicerates, and even panarthropods (tardigrades and onychophorans), has shown that the overall expression and function of Hox genes is likely conserved (reviewed in [29, 35, 56, 69]). Body segmentation and formation of tagmata, however, are not entirely under control of the Hox genes. The anterior head region of *Drosophila* for example is under control of a different gene regulatory network including the so-called head gap genes (e.g. [12, 23, 74]).

Two other genes that are involved in the determination of body regions in *Drosophila* are the homeodomain encoding gene *teashirt* (*tsh*) [18], and the zinc finger encoding gene *spalt* (*sal*) [20, 38]. Similar to the expression of Hox genes in broad domains along the AP body axis, *tsh* is expressed in the entire trunk region of the embryo, and mutation of this gene leads to the disruption of the entire trunk region of the embryo [18]. It has further been shown that *tsh* interacts with trunk Hox genes, and that it represses characteristics of anterior segments in the trunk, making it an essential factor of trunk identity [3, 16, 62]. Similarly, *tsh* is regulated by gap genes to specify segments [64], and is involved in providing leg-identity to trunk appendages [24]. In *Drosophila*, and its closest relatives, there is a second paralog of this gene, called *tiptop* (*tio*) [41] that shares some of the functions of *tsh* [7]. In all other previously investigated arthropods, however, there is only one ortholog of *tio* and *tsh*, called *tiptop/teashirt* (*tio/tsh*) (e.g. [45]). Investigation of the *tio/tsh* gene expression in other arthropods than *Drosophila* revealed widely conserved expression suggesting that the overall function of *tio/tsh* as a trunk-regulator gene may be conserved in arthropods as a whole [24, 45, 55, 68].

The anterior and the posterior borders of *tsh*-expression in *Drosophila* are under control of one of the two spalt genes, *spalt-major* (*salm*) [38, 40, 60, 62]. Corresponding with the repressive function of *salm* on *tsh*, the expression profiles of these genes are complementary in *Drosophila* with *salm* being expressed anterior and posterior adjacent to *tsh* [40, 62]. In the beetle *Tribolium castaneum*, however, this correlation is not preserved, suggesting that the single *spalt* (*sal*) gene in this species is not regulating *tsh* expression [6, 68, 72]. Whether the situation in the fly, or the beetle is ancestral, however, has not been investigated.

In this study, we expand investigation of *tsh* gene expression to Onychophora, and thus Panarthropoda, and provide data on another myriapod model species, the common pill millipede *Glomeris marginata*. We also, for the first time, investigate early expression patterns of *tsh* in a spider. Finally, we conducted a comprehensive analysis of *spalt* (*sal*) gene expression in several arthropod species and an onychophoran to investigate the potential interaction of *sal* and *tsh* genes in Panarthropoda.

## Methods

### Animal husbandry and fixation of embryos

Embryos were obtained, embryonic membranes were removed, and embryos were treated for subsequent in situ hybridization experiments, as described in Janssen et al. [30] (*Glomeris*), Prpic et al. [58] (*Parasteatoda tepidariorum*), Schinko et al. [65] (*Tribolium*), and Hogvall et al. [25] (*Euperipatoides kanangrensis*). Developmental stages were defined as per Janssen et al. [30] (*Glomeris*), Mittmann and Wolff [47] (*Parasteatoda*), Strobl and Stelzer [70] (*Tribolium*), and Janssen and Budd [34] (*Euperipatoides*).

### RNA extraction, gene cloning, whole mount in situ hybridization, and nuclear staining

Total RNA or messenger RNA was isolated from embryos of mixed stages of *Tribolium*, *Parasteatoda*, *Glomeris* and *Euperipatoides,* respectively, using TRIZOL (Invitrogen). RNA was reverse transcribed into cDNA using the SuperScript First Strand kit (Invitrogen). Gene fragments were amplified by means of RT-PCR with gene-specific primer-based sequence information from sequenced embryonic transcriptomes of *Glomeris* and *Euperipatoides*, and published genomes of *Parasteatoda* and *Tribolium*. Nested PCRs were run with internal primers, using 1 µl of first PCR-product as template. All used primer sequences are listed in Additional file 5: Table S1. Investigated gene fragments were cloned into the PCRII vector (Invitrogen) and sequenced on an ABI3730XL automatic sequencer (Macrogen, Seoul, South Korea). Gene identifiers are listed in Additional file 6: Table S2. One-colour in situ hybridizations were performed as described in Janssen et al. [37], and two-colour in situ was performed as per Janssen et al. [32] using a digoxigenin- (for one gene) and a fluorescein-labelled (for the second gene) probe in parallel. We detected the first gene with the digoxigenin-labelled probe with BM-Purple (Roche) (blue signal). After that, the antibody was removed with 0.1 M glycine, pH = 2.0 (10-min incubation at room temperature). We then detected the second probe with SIGMAFAST Fast Red TR/Naphthol AS-Mx (Sigma) (red signal).

Cell nuclei were stained incubating embryos in either 3 μg/ml of 4-6-diamidino-2-phenylindole (DAPI) or in SYBR Green (Invitrogen) in phosphate buffered saline with 0.1% Tween-20 (PBST-0.1%) for 20 min at room temperature. Excessive DAPI/SYBR Green was removed by several incubation steps in PBST-0.1%.

### Phylogenetic analysis

We identified potential orthologs by performing reciprocal BLAST searches against the sequenced embryonic transcriptomes of the millipede *Glomeris* and onychophoran *Euperipatoides* and published genomic sequences of the beetle *Tribolium* and the spider *Parasteatoda*. In these BLAST searches, we used the *tiptop/teashirt* ortholog of *Parasteatoda tepidariorum*, and the *Drosophila melanogaster spalt* ortholog as baits.

Amino acid sequences of the complete coding regions of putative *tio/tsh* and *sal* orthologs, and outgroup genes [the *Drosophila Zinc finger homeodomain 1* (*Zfh1*) for *tio*/*tsh*, and *Krüppel* orthologs (for *sal*)], were aligned using T-Coffee followed by manual editing in SeaView [22, 48] using default parameters in MacVector v12.6.0 (MacVector, Inc., Cary, NC). For both genes, Bayesian phylogenetic analyses were executed using MrBayes [26] with a fixed WAG amino acid substitution model with gamma-distributed rate variation across sites (with four rate categories, unconstrained exponential prior probability distribution on branch lengths, and exponential prior for the gamma shape parameters for among-site rate variation. Topologies of the trees were calculated applying 300,000 cycles for the Metropolis-Coupled Markov Chain Monte Carlo (MCMCMC analysis (four chains, chain-heating temperature of 0.2. Markov chains were sampled every 200 cycles. Default settings were used, defining 25% of the samples as burn-in information. Clade support was calculated with posterior probabilities in MrBayes. Sequence identifiers are listed in Additional file 6: Table S2. Zinc finger motives have been identified manually.

### Data documentation

Bright field microscopy and visualization of DAPI- and SYBR green-stain was executed using a Leica DC490 digital camera equipped with a UV light source mounted onto a MZ-FLIII Leica dissection microscope. When appropriate, linear adjustments were made on colour contrast and brightness using the image-processing software Adobe Photoshop CS6 for Apple Macintosh (Adobe Systems Inc.).

## Results

### Sequence analysis

**Fig. 1:**
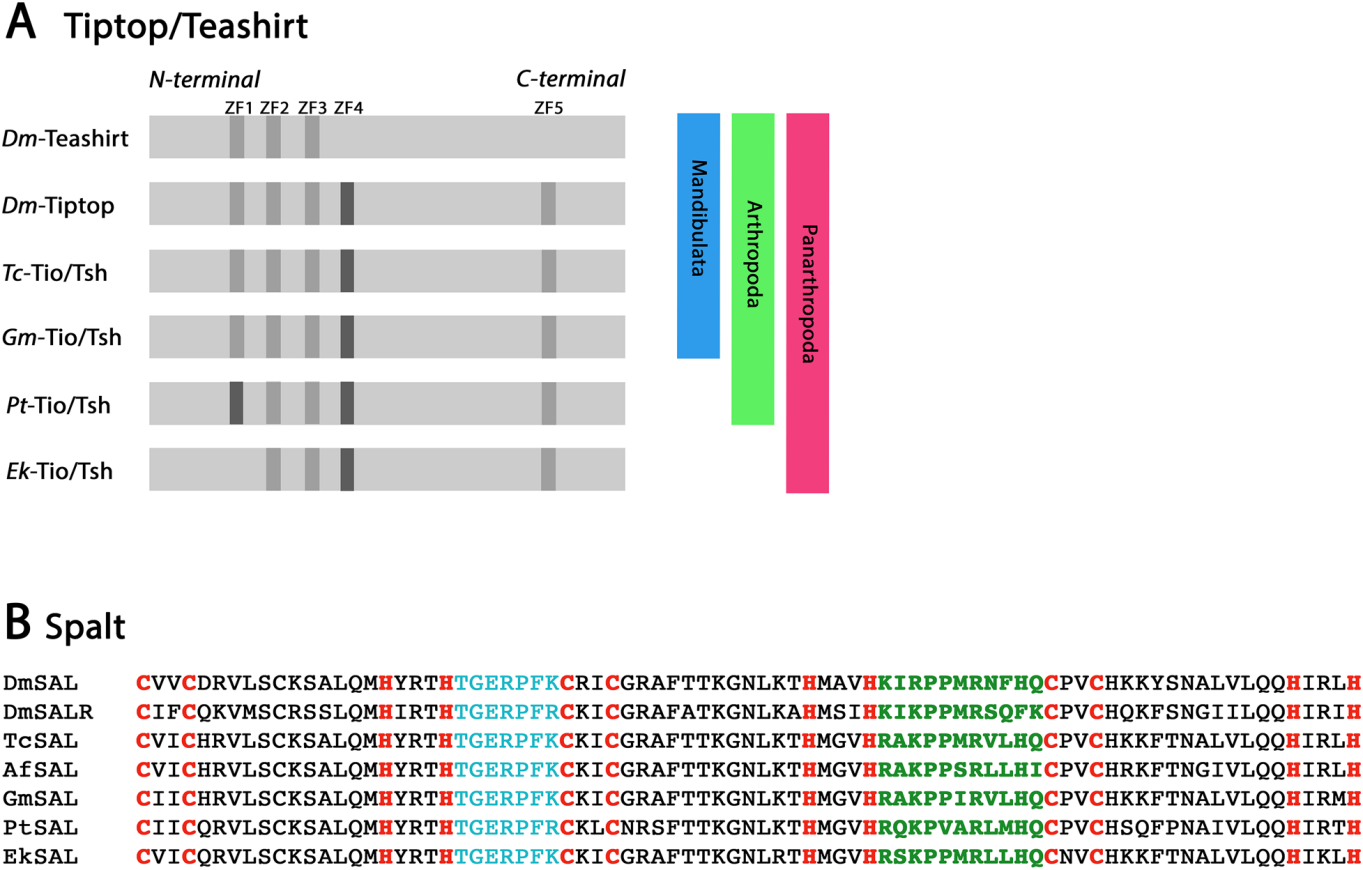
**A** Distribution of zinc fingers (ZF) in the protein coding region of Teashirt, Tiptop, and Tiptop/Teashirt orthologs. The protein is depicted in light grey, “regular” ZFs are in darker grey, and “derived” ZFs are in darkest grey. Note that distribution, number and kind of ZFs are conserved between *Drosophila* Tiptop and mandibulate Tiptop/Teashirt genes, while the complement of *Drosophila* Teashirt is clearly derived from this pattern. In the spider, additionally, the first ZF is derived and in the onychophoran, the first ZF is missing. **B** Sequence comparison of a characteristic region of Spalt and Spalt-related orthologs. Note that they possess a variable number of ZFs, but that they all share a unique combination of ZFs. One N-terminal ZF is linked by seven amino acids (marked in blue) to a downstream second ZF that is linked by a unique number of 11 amino acids (marked in green) to a third ZF. This combination and linking of ZFs is unique for Spalt proteins. Species abbreviations: Dm, *Drosophila melanogaster* (Insecta: Diptera); Ek, *Euperipatoides kanangrensis* (Onychophora); Gm, *Glomeris marginata* (Myriapoda); Pt, *Parasteatoda tepidariorum* (Chelicerata), and Tc, *Tribolium castaneum* (Insecta: Coleoptera)

In all hitherto investigated panarthropods, there is only one *tiptop*(*tio*)/*teashirt*(*tsh*)-type gene, except for *Drosophila* that possesses two paralogs, *tsh* and *tio* [24, 41, 45, 55, 68]. Tio- and Tsh-type proteins can be identified by the presence of a unique set of five zinc fingers (ZFs) (Fig. 1A) [10]. Four of these ZFs are located N-terminally, while the fifth ZF is located in the C-terminal region of the protein (Fig. 1A). The ZF2, ZF3 and ZF5 are of the C_2_H_2_-type, while ZF4 is of the C_2_HC-type (Fig. 1A). In a phylogenetic analysis, all Tio/Tsh genes cluster together (Additional file 1: Figure S1).

The *Drosophila* Teashirt protein only possesses three ZFs (ZFs 4 and 5 are missing); Teashirt thus represents a derived paralog. The onychophoran Tio/Tsh protein lacks ZF1, while in the spider this ZF is of the C_2_HC-type instead of the C_2_H_2_-type as in all other here investigated arthropods (Fig. 1A).

Spalt (Sal) proteins possess a variable number of ZFs, but all share a unique combination of ZFs [40]: one N-terminal ZF is linked by seven amino acids to a downstream second ZF that is linked by a unique number of 11 amino acids to a third ZF (Fig. 1B. This combination and linkage of ZFs appears to be unique for Spalt proteins, especially the 11-amino acid link; the seven-amino acid link is more common for ZF proteins (e.g. found in Krüppel proteins) (e.g. [66]). In a phylogenetic analysis, Sal proteins cluster together and separate from Krüppel proteins, the most similar but distantly related zinc finger proteins (Additional file 2: Figure S2).

### Embryonic expression patterns of panarthropod *tiptop*/*teashirt* (*tio*/*tsh*) genes

**Fig. 2:**
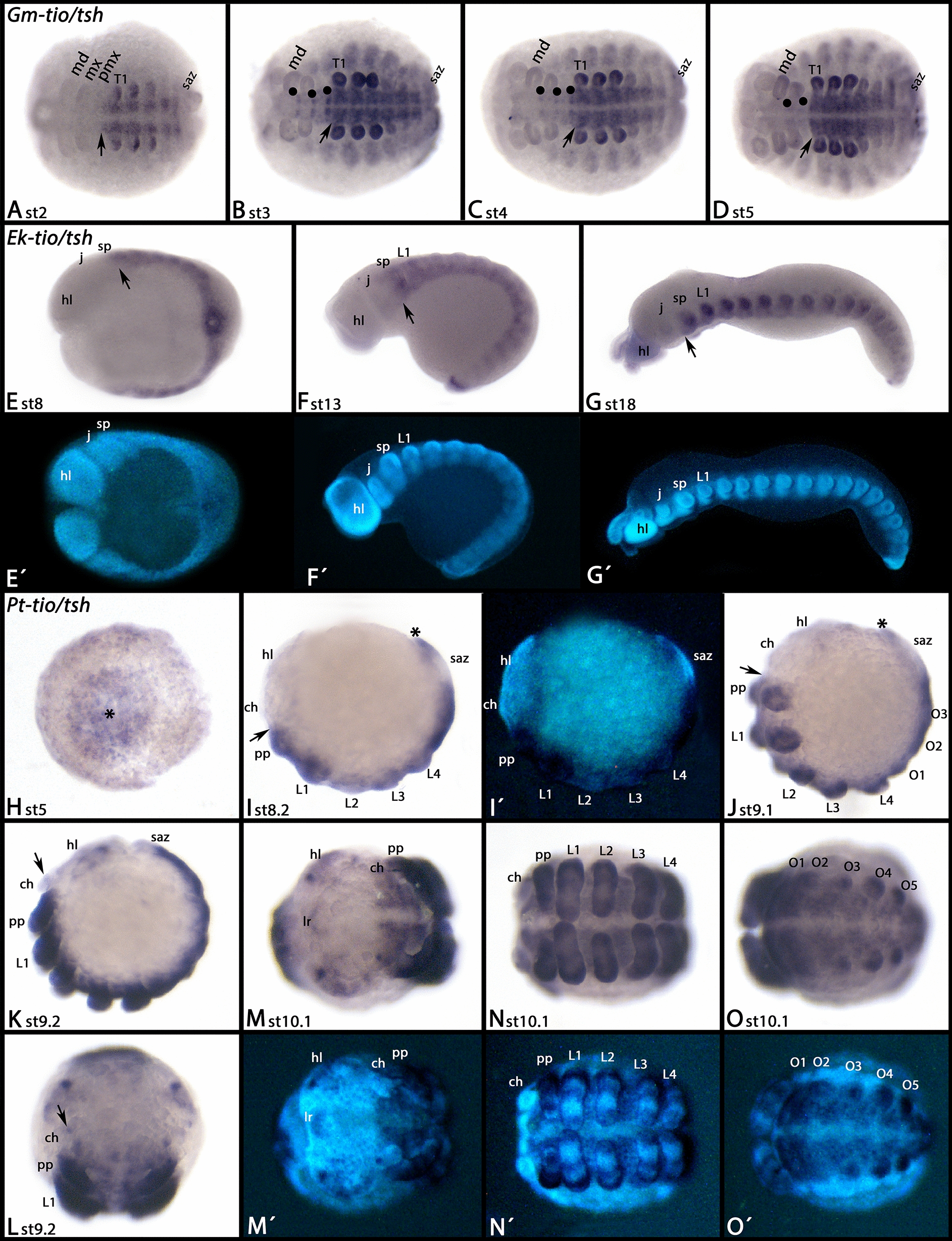
Expression of *tiptop/teashirt* (*tio/tsh*) in *Glomeris* (**A**–**D**), *Euperipatoides* (**E**–**G**), and *Parasteatoda* (**H**–**O**). In all panels, anterior is to the left except for panels J and L (anterior up). All panels show ventral views, except for **E**–**G**, **I**–**K** (lateral views). **E**´–**G**´, **I**´, and **M**´–**O**´ represent DAPI staining of the embryos shown in the corresponding panels. Arrows in **A**–**G** and **I**–**K** point to the anterior border of expression. Note that there is no expression anterior to that, except for some staining in the nervous system (filled circles in **B**, **C**, and arrow in **L**). The asterisk in panel H marks the centre of the germ disc, the future posterior pole of the embryo. Asterisks in **I**, **J** mark expression in the posterior of the SAZ. *ch* chelicera, *hl* head lobe, *j* jaw, *L1* first walking-limb bearing segment, *md* mandible, *mx* maxilla, *O1* first opisthosomal segment, *pmx* postmaxillary segment, *pp* pedipalp, *SAZ* segment addition zone, *sp* slime papilla, *T1* first trunk segment

*Glomeris tio/tsh* is expressed in all segments posterior to the postmaxillary segment (Fig. 2A). Expression is enhanced in segmental blocks on either side of the ventral midline, tissue that most probably contributes to the developing ventral nervous system, as well as in the buds of the outgrowing legs (Fig. 2A). Later, expression also occurs in the outgrowing dorsal segmental units and in smaller segmental neurogenic patches in the postmaxillary segment, the maxillary segment and the mandibular segment (Fig. 2B, C). These dots eventually weaken and disappear, but the strong expression in the complete trunk remains (Fig. 2D).

**Fig. 3:**
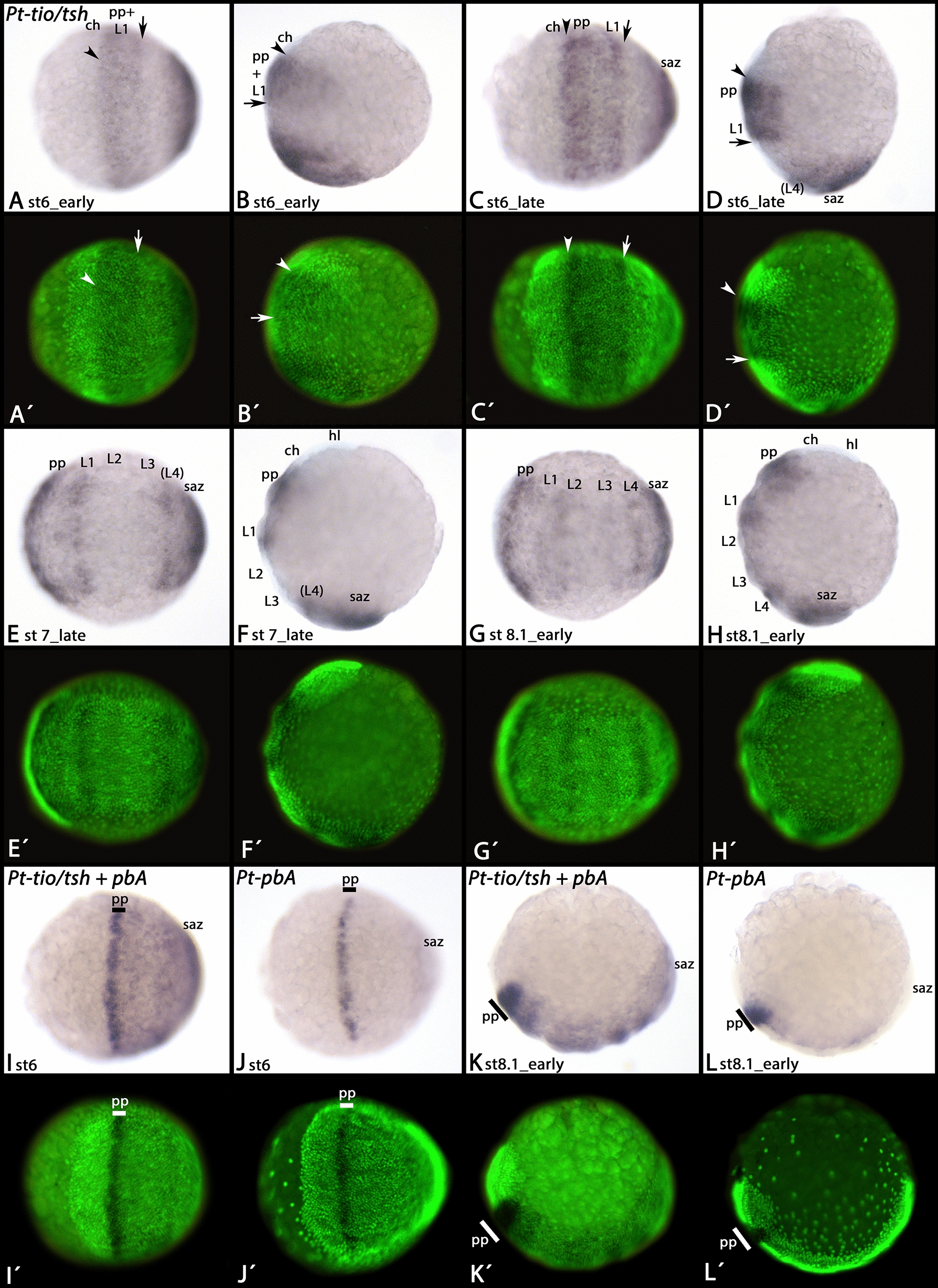
Early expression of *Parasteatoda tiptop/teashirt.* **A**, **C**, **E**, **G**, **I** and **J** represent ventral views and anterior to the left. **B**, **D**, **F**, **H**, **K** and **L** represent lateral views and anterior up. **A**´–**L**´ represent SYBR Green staining of the embryos shown in the corresponding **A**–**L**. Arrowheads mark the most anterior expression of *tio/tsh*. Arrows mark the most posterior extension of the anterior domain of expression. Bars in **I**–**L** indicate the position of the pedipalp-bearing segment that strongly expresses the anterior Hox gene *proboscipedia-A* (*pbA*). Note that there is no expression of *tio/tsh* anterior to the expression of *pbA* (**I**–**L**). Abbreviations as in Fig. 2

*Euperipatoides tio/tsh* is expressed in all tissue posterior to the jaw-bearing segment (Fig. 2E–G). Expression of the single *tio/tsh* gene of the spider *Parasteatoda* has partially been described by March et al. [45]. However, in their work they only describe late developmental stages, and some aspects of expression at these stages have not been recognized or described. In our analysis, we include early developing stages. Earliest expression is in the form of a patch in the centre of the germ disc (Fig. 2H), and a broad ring of expression close to the margin of the disc (Figs. 2H and 3A, B). Careful observation of expression in closely staged embryos reveals that the anterior border of this expression is between the cheliceral and the pedipalpal segment (Figs. 2I, J and 3A–L). Monochromatic double in situ hybridization with *tio/tsh* and the anterior Hox gene *proboscipedia-A* (*pbA*) [67] shows that there is no expression of *tio/tsh* anterior to the very strong (dominant) expression of *pbA* in the pedipalpal segment (Fig. 3I–L). Dichromatic double in situ hybridization, as used for *spalt* and *pbA* (see below) did not work for *tio/tsh* because of its relatively low level of expression. The posterior border of the early gap-gene like domain of *tio/tsh* is between the first and second walking-leg bearing segment (Fig. 3A–H). The central domain (future posterior of the embryo) is representing expression in the segment addition zone (SAZ) and the newly forming fourth walking-limb bearing segment (Fig. 3D–H). At subsequent developmental stages, expression also appears in the second and third walking-leg bearing segment (Figs. 2I–K and 3G, H). At stages 8.2 and 9.1, expression is in the posterior of the SAZ (or near its posterior margin) (Fig. 2I, J), but at stage 9.2, the posterior of the SAZ is free from expression, suggesting dynamic expression of *tio/tsh* in the SAZ (Fig. 2K). From stage 9.2 onwards, punctate expression appears in the head lobes and the chelicerae (Fig. 2K–M). In stage 10.1 embryos, expression in the pedipalps and the legs is in the form of stripes of enhanced expression (Fig. 2N. In the opisthosoma, expression is restricted to ventral tissue including the opisthosomal appendages; the dorsal margins of the opisthosoma do not express *tio/tsh* (Fig. 2O).

### Embryonic expression patterns of panarthropod *spalt* (*sal*) genes

**Fig. 4:**
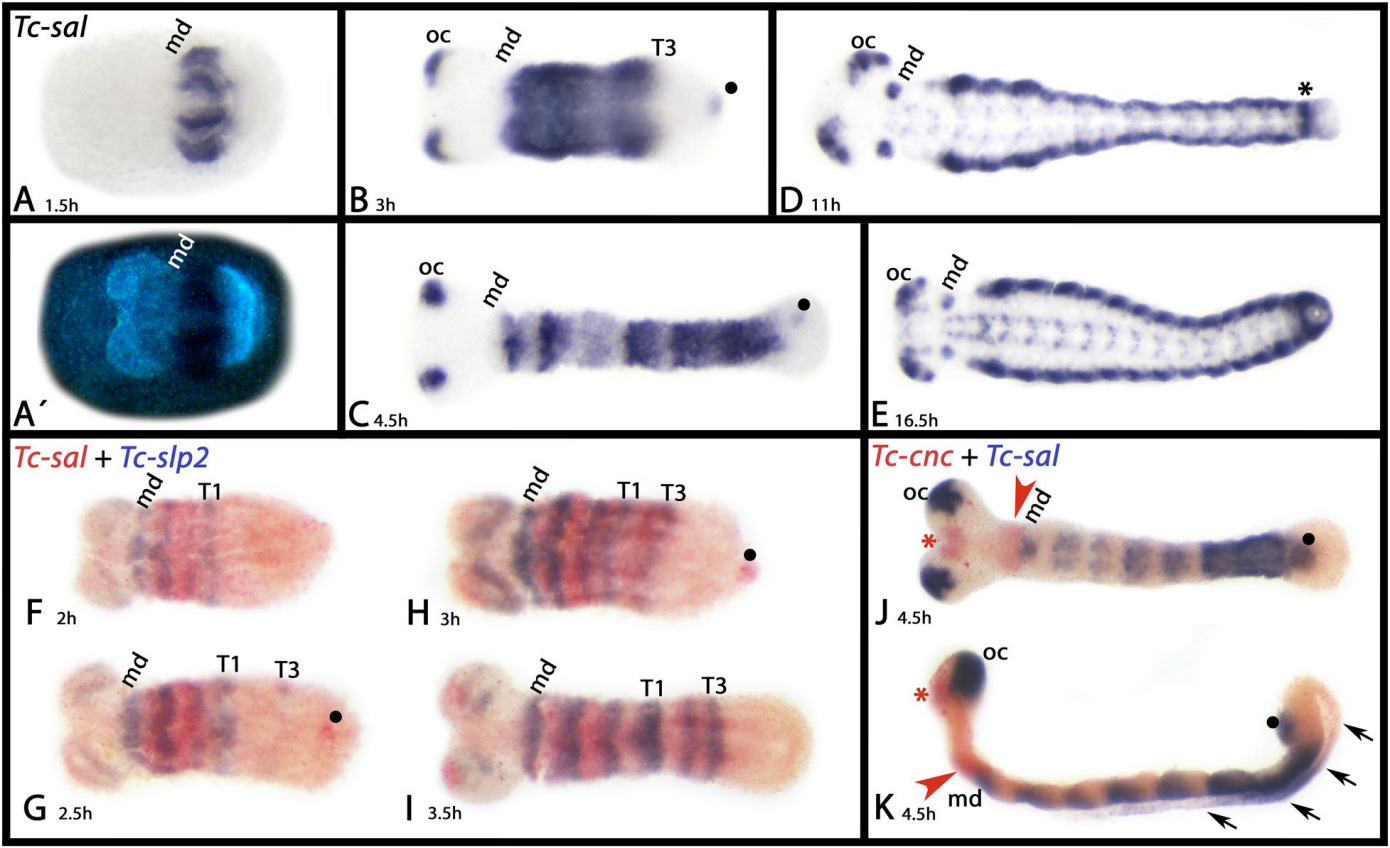
Expression of *Tribolium spalt*. In all panels, anterior is to the left, ventral views (except **K**, lateral view). **A**´ represents a DAPI staining of the embryo shown in **A**. Filled circles in **B**–**D**, **H**, **J** and **K** mark expression in an internal structure at the posterior end of the embryo. The black asterisk in **D** marks segmentation gene like expression in the last-formed segment. Red asterisks in **J** and **K** mark the anterior expression domain of *cap-n-collar* (*cnc*) (the cap). The red arrowheads mark the posterior domain of *cnc* (the collar). Arrowhead in **K** point to expression in the amnion. Abbreviations as in Fig. 2; *oc* ocular region, *T* thoracic segment

*Tribolium sal* has been the subject of earlier investigations. However, most of this work focused on either the role of *sal* in tracheal development or development of the elytra, and thus the focus of these studies was on late developmental stages [15, 72]. Expression of *Tribolium sal* in a relative early developmental stage was provided by Shippy et al. [68] (in their Additional files 3, 4) and a comprehensive analysis was provided by Berghammer ([6], doctoral thesis in German). Here we investigate the complete embryonic expression profile of *sal*, including the earliest stages of expression, verifying the expression patterns as described by Berghammer [6]. The gene is first expressed in the form of a broad gap-gene like domain (Fig. 4A–C). Double-staining with the conserved forkhead transcription factor encoding gene *sloppy-paired* (*slp*) [11] and the secondary head gap gene *cap-n-collar* (*cnc*) [14] reveals the position of the anterior border of this domain: *sal* is expressed posterior to the mandibular segment (Fig. 4F–K) (cf. Berghammer [6] who comes to the same conclusion using different genetic markers). From there, expression extends posteriorly throughout the complete germ band, except for the posterior of the SAZ (Fig. 4A–C, F–I). At later developmental stages, however, the gap-gene like expression transforms into a transient metameric pattern similar to that of pair-rule and segment-polarity genes (Fig. 4C, J, K). Towards the end of germ band extension, only the tissue anterior to the SAZ expresses *sal* in the form of a solid, albeit small, segmentation-gene like domain (Fig. 4D).

Additional expression of *Tribolium sal* is in the ocular region (Fig. 4B–K), the ventral nervous system (Fig. 4D, E), and an internal structure at the very posterior of the developing embryo (Fig. 4B, C, G, H, J, K). The gap-gene like domain of expression is also present in the dorsal epithelium (Fig. 4K) [5].

**Fig. 5:**
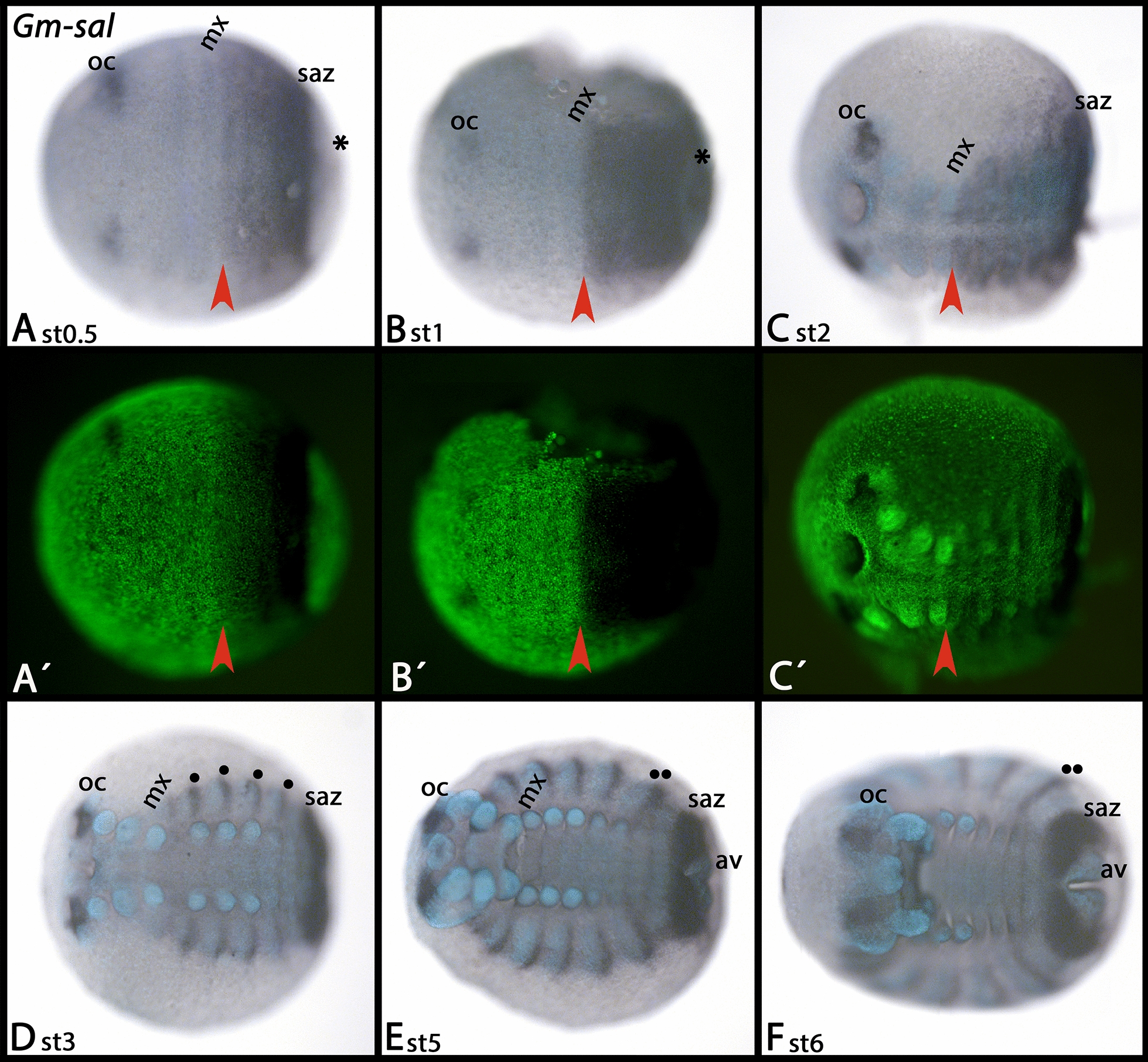
Expression of *Glomeris spalt*. In all panels, anterior is to the left, ventral views. **A**´–**C**´ represent SYBR Green staining of the embryos shown in **A**–**C**. Red arrowheads mark the most anterior border of the broad “trunk” domain of expression; note that expression is also in the ocular region (oc). Filled circles in **D**–**F** mark expression in the dorsal segmental units; note the double filled-circles in **E** and **F** that mark two fused domains of expression (cf. [33]). Abbreviations as in Fig. 2

*Glomeris sal* is expressed in the ocular region and in all tissue posterior to the maxillary segment, except for tissue posterior to the SAZ that gives rise to the anal valves (Fig. 5A–C). While expression in the ocular region and the SAZ remains throughout further development, at stage 3, expression in the trunk disappears except for stripes in the dorsal segmental units (Fig. 5D–F).

There are two *spalt* paralogs in the spider *Parasteatoda* that display fundamentally different expression patterns suggesting neo-functionalization of these genes after their likely duplication somewhere in the lineage leading to Arachnopulmonata [67].

**Fig. 6:**
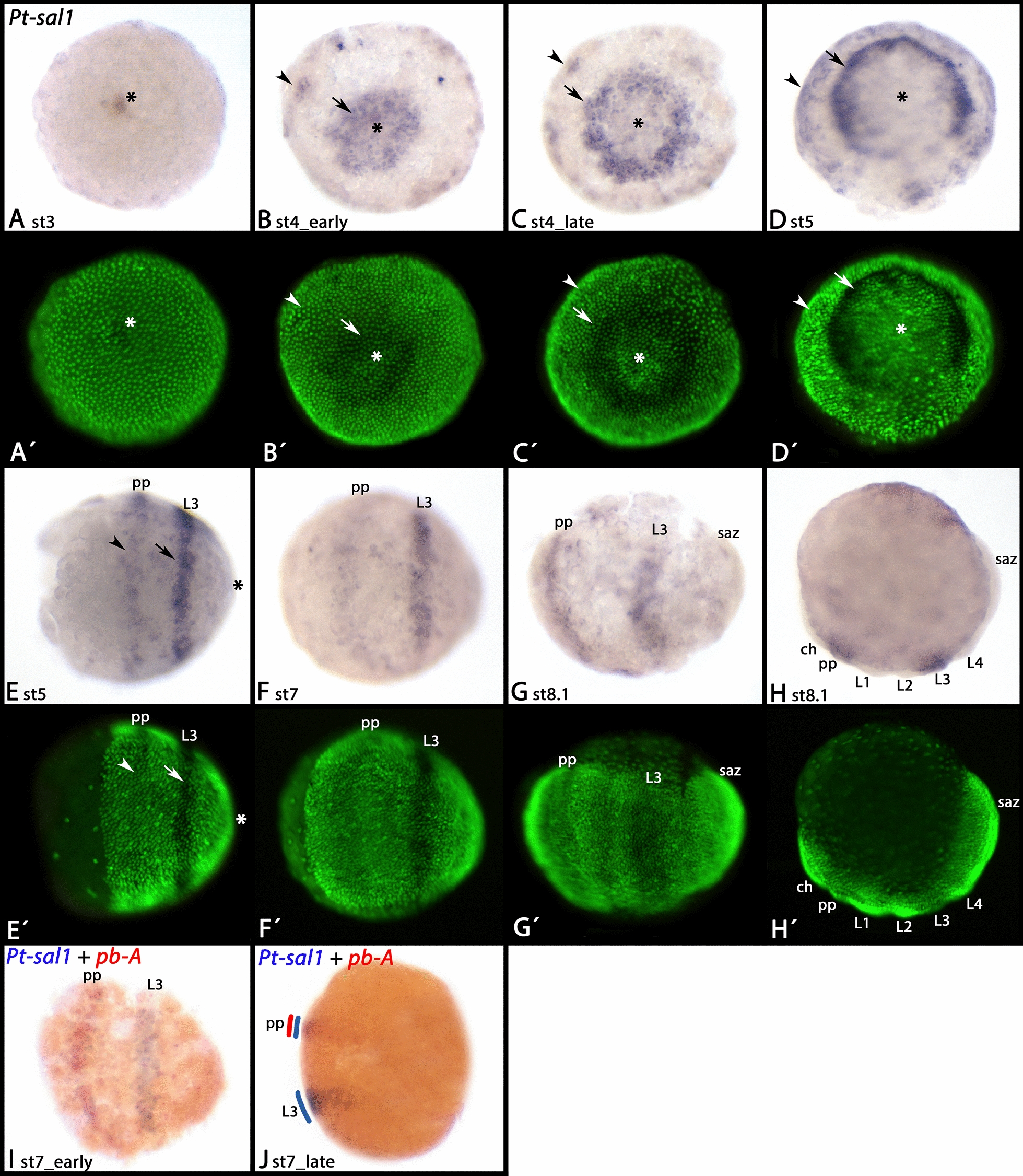
Early expression of *Parasteatoda spalt1*. **A**–**D** show the germ disc. The asterisks mark the centre of this disc, the later posterior pole of the embryo (cf. **E**). In all panels, the arrows point to the transforming central domain if expression and the arrowheads point to the more distal (later anterior) domain of expression. **E**–**H** show the early germ band, ventral views (except for **H**, lateral view). In **E**–**I**, anterior is to the left. In **J**, anterior is up, lateral view. **A**´–**H**´ represent SYBR Green-stained embryos as shown in **A**–**H**. Abbreviations as in Fig. 2

**Fig. 7:**
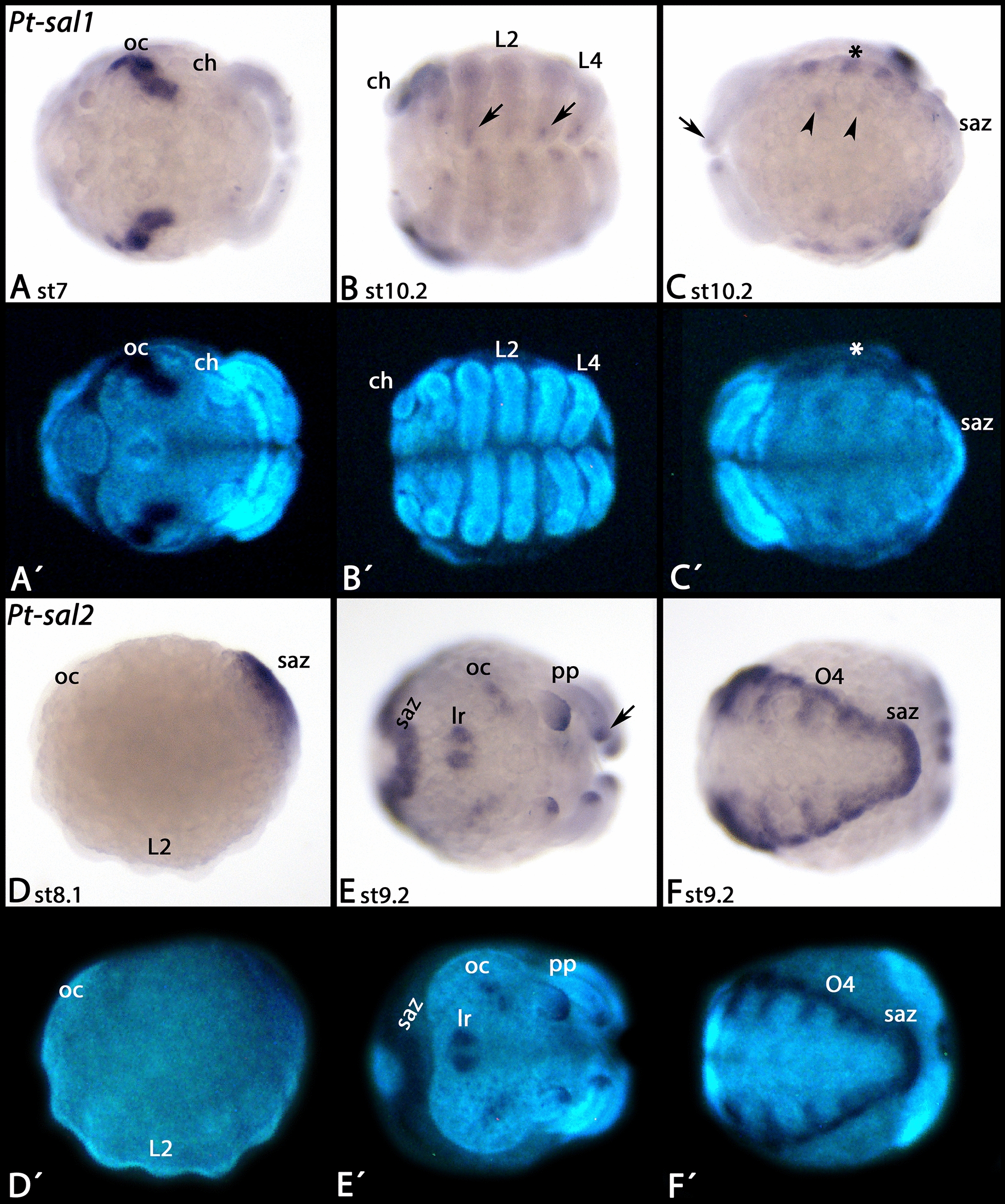
Late expression of *Parasteatoda spalt1* and expression of *Parasteatoda spalt2*. In all panels, anterior is to the left. Ventral views, except **D** (lateral view). **A**´–**F**´ represent DAPI staining of the embryos shown in **A**–**F**. Arrows in **B** and **C** point to expression in the tips of the legs. Arrowheads in **C** point to expression in the developing book lungs and tracheal lungs. The asterisk marks expression in the dorsal region of the opisthosoma. The arrow in **E** points to expression in the tips of the legs. Abbreviations as in Fig. 2; lr, labrum

*Parasteatoda sal1* is first expressed in the form of a small domain in the centre of the germ disc (Fig. 6A). This domain then broadens, and an additional ring forms close to the periphery of the disc (Fig. 6B). Then expression disappears from the centre of the former domain resulting in a second ring of expression (Fig. 6C–E). Double-staining with the Hox gene *proboscipedia-A* (*pb-A*) [67], and following the stripes throughout development until morphological segmental landmarks form, reveals that the anterior (peripheral) ring corresponds with the pedipalpal segment, and the posterior (central) ring with the third leg-bearing segment (Fig. 6F–J). Later, this expression disappears, and de novo expression is seen in the ocular region, the tips of all appendages (except for the chelicerae and the labrum), the developing book lungs and tracheal lungs in opisthosomal segments two and three, and in the form of metameric spots along the dorsal of the opisthosoma (Fig. 7A–C).

Expression of *Parasteatoda sal2* starts later than that of *sal1* and in the form of a solid posterior domain (Fig. 7D). Additional expression is in the tips of the prosomal appendages except for the chelicerae, the labrum, the ocular region (albeit weaker than *sal1*), and along the dorsal of the opisthosoma (Fig. 7E, F).

**Fig. 8:**
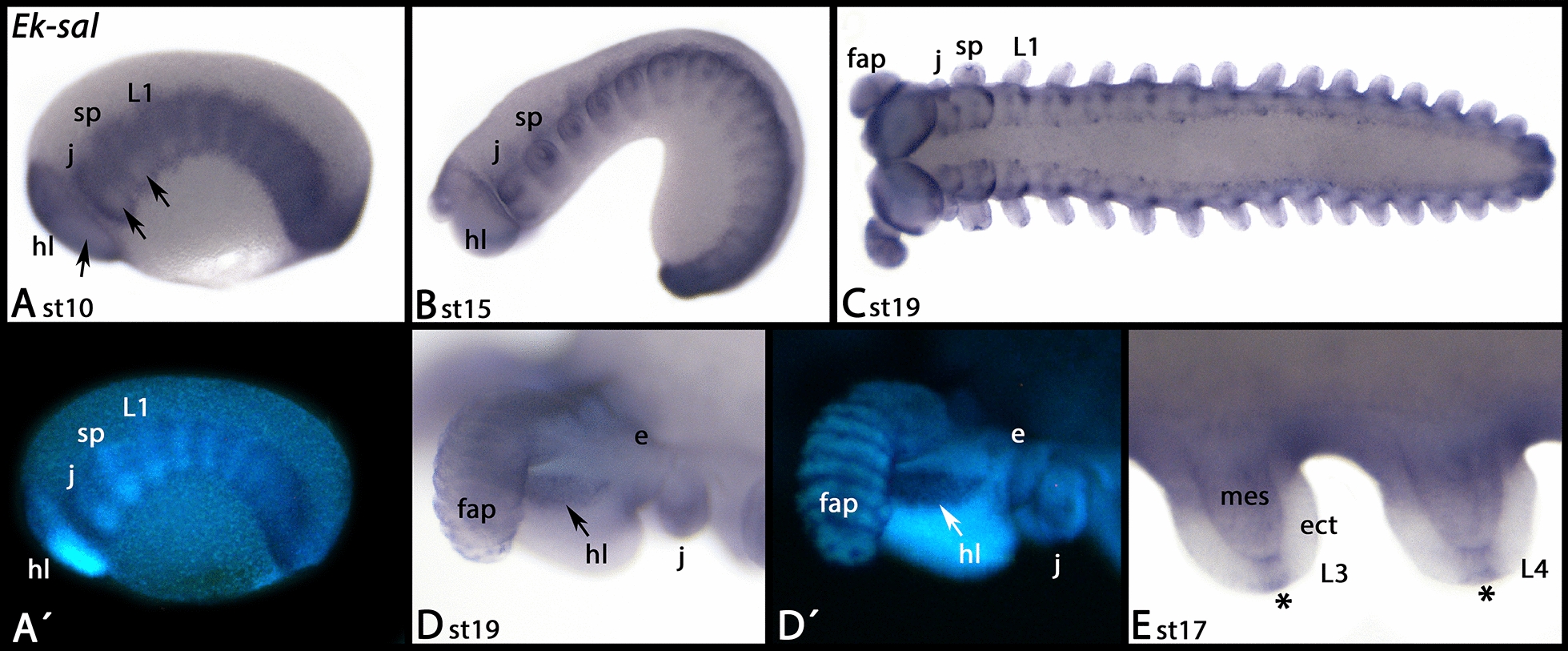
Expression of *Euperipatoides spalt*. In all panels, anterior is to the left, lateral views, except **C** and **E** (ventral views). **A**´ and **D**´ represent DAPI staining of the embryos shown in **A** and **D**. Arrows in **A** point to lack of expression in the ventral region. The arrow in **D** points to expression in the developing brain. Asterisks in **E** mark expression in the tips of the legs. Abbreviations as in Fig. 2; *e* eye, *ect* ectoderm, *fap* frontal appendages (= antennae), *mes* mesoderm

*Euperipatoides sal* is first expressed in all tissue except for the region where the limb buds grow out and the centre of the head lobes (Fig. 8A). Later, *sal* is expressed along the dorsum of the complete embryo, while there is only faint or no expression in ventral tissue (Fig. 8B). At late developmental stages, expression appears in the tips of the limbs, the most proximal tissue of the limbs, and the limb-mesoderm (Fig. 8C, E), the brain (Fig. 8C, D), and in the form of metameric patches in the ventral tissue of the trunk (Fig. 8C).

## Discussion

### *Gene expression suggests a function of tiptop*/*teashirt orthologs as panarthropod “trunk” selector genes*

The term “trunk” is used in a somewhat arbitrary way for arthropods, but usually refers to the body unit that bears the less-modified (often) locomotory appendages, a feature that distinguishes the “trunk” from the “head”, another arbitrary body unit that is defined by the presence of highly modified sensory and food-processing appendages (e.g. [4, 17, 27].

A group of genes that is in control of segment-identity and possibly also tagmosis are the famous and highly conserved Hox genes (e.g. reviewed in [29, 35, 56, 69]. It has been suggested that the “head” and the “trunk” tagmata could be under control of different sets of Hox genes, but the shifting expression domains of Hox genes in different groups of arthropods are not strictly aligned with these tagmata (e.g. [29]). However, in *Drosophila*, it has been shown that the Hox genes interact with *tsh* that serves as a co-factor of the former. Together, they repress head-identity in the trunk [2, 16, 61, 62, 71]. One conserved function of *tio/tsh* genes appears to be the discrimination between highly modified anterior “head” segments, and more posterior (and less modified) “trunk” segments, as recently highlighted by March et al. [45].

We believe however, that the study of March et al. [45] suffers from some technical limitations that may have led the authors to somewhat misinterpret and over-simplify their data. The centipede expression data provided in March et al. [45] are rather unclear, but the statement that *tio/tsh* is in all limb buds except for the mandibles, is not supported by our millipede data. The statement that expression of *tio/tsh* in insects is restricted to the thoracic segments is an over-simplification. Clearly, in *Tribolium tio/tsh* is expressed at comparable level in thoracic and abdominal segments [68]. Unfortunately, expression data of *tio/tsh* in the milkweed bug *Oncopeltus fasciatus* are of rather low quality; background staining is hardly distinguishable from specific signal in the segments [24, 45], Additional files 3, 4). Expression in the firebrat *Thermobia domestica* is not restricted to the thorax either, but extends (albeit at low level) into the abdominal segments (stronger in the first abdominal segment) [55].

As March et al. [45] pointed out, correlation between Hox genes and *tio/tsh* genes in the arthropod species they investigated must be different from that in *Drosophila* [45], and indeed, we could not find any conserved correlation between the expression patterns of Hox genes and *tio/tsh* in our research organisms either. Correlation between the Hox co-factor *disconnected* (*disco*) [43, 64] and *tio/tsh* [61] does not appear to be conserved in panarthropods outside *Drosophila* either [36, 52], this study), suggesting that the entire network of head versus trunk patterning downstream of *tio/tsh* in these arthropods is significantly different from that in *Drosophila*.

Data from other bilaterian animals such as mice and flatworms suggest that *tsh*-like genes may play an ancestral function in head versus trunk development [44, 51], discussed in [45]. However, even the available functional data on insect *tio/tsh* (the only available functional data in arthropods) are not conclusive regarding a conserved homeotic function, which may be because of limited penetrance of RNAi-mediated knock-down of *tio/tsh* in *Oncopeltus* and *Tribolium* (discussed in [45]).

In any case, our gene expression data reveal a clear association of *tio/tsh* with the development of walking-leg type appendages. In the onychophoran, these are the walking limbs (lobopods) and the slime papillae, that are modified walking limbs that must have evolved after terrestrialization somewhere in the Devonian or Carboniferous period (e.g. [21, 63]). In the slime papillae, the nephridial organs evolved into salivary glands (e.g. [46]). The more derived onychophoran jaws and frontal appendages, however, do not express *tio/tsh*. In the myriapod, all walking leg-bearing segments and the walking limbs express *tio/tsh*, but neither of the head appendages (maxillae, mandibles, labrum, antennae) nor (except for secondary ventral expression in the nervous system) their corresponding segments. In the centipede *Lithobius atkinsoni*, the forcipules (poison fangs) which represent modified walking limbs as well, still express *tio/tsh* [45]. In the spider, walking legs and pedipalps express *tio/tsh*, are patterned very similarly, only show little morphological differences (especially in more primitive spiders), and are *inter alia* used for walking (e.g. [1, 19, 53, 57, 73], reviewed in [54]). The situation in spiders however also represents an exception because *tio/tsh* is expressed in the opisthosomal limb buds that represent highly modified appendages such as the book lungs and the spinnerets (reviewed in [54]). In the opisthosoma of spiders, one (or more) of the posterior Hox genes that are exclusively expressed in this body region may repress the development of walking-leg type appendages, even in the presence of *tio*/*tsh* [67].

**Fig. 9:**
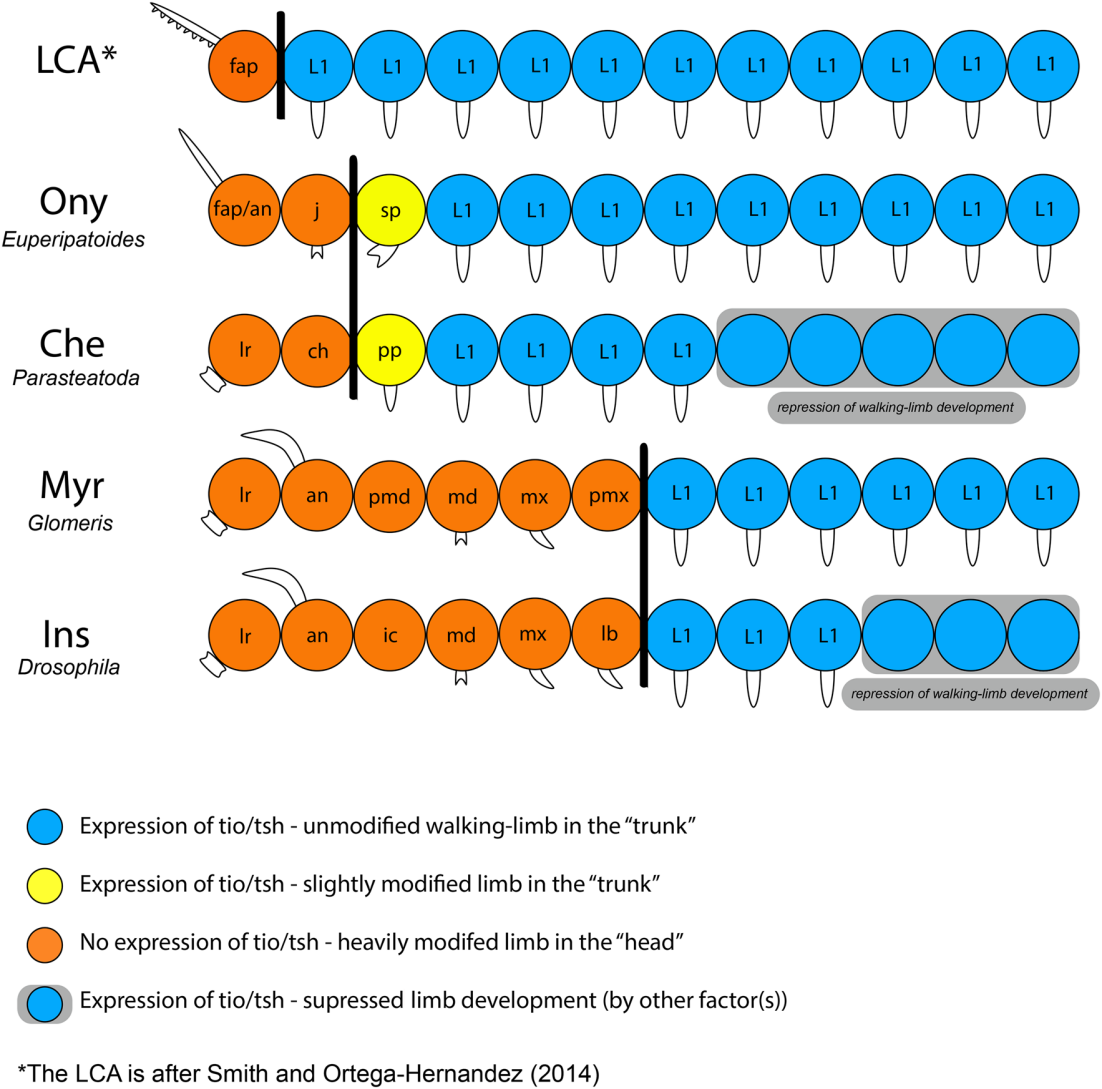
Correlation of *tio/tsh* expression, appendage-types, and tagmosis in panarthropods and the predicted last common ancestor (LCA) of arthropods and lobopodians. The vertical black lines represent the border between the “head” carrying highly modified appendages (orange), and the “trunk” carrying walking-limb type appendages (blue and yellow). Shown is the situation as predicted for the last common ancestor (LCA), the onychophoran *Euperipatoides* (Ony), the chelicerate *Parasteatoda* (Che), the myriapod *Glomeris* (Myr), and the insect *Drosophila* (Ins). Abbreviations as in Fig. 2, and *fap/an*, the onychophoran frontal appendage (a functional antenna), *ic* intercalary limb-less segment of insects, *lb* labium, *lr* labrum, *pmd* premandibular limb-less segment of myriapods

The available panarthropod expression data clearly suggest a role of *tio/tsh* in the development of walking-limb type “trunk” appendages. Highly derived “head” appendages, however, do not express *tio/tsh*, while all walking-limbs (legs) and walking-limb like appendages such as the pedipalps of spiders, express *tio/tsh* (summarized in Fig. 9). From an evolutionary point of view, we suggest that the expression domain of *tio/tsh* must have shifted towards posterior as more anterior segments got specialized and incorporated into the “head”. In the last common ancestor of arthropods and onychophorans, likely only the most anterior “frontal appendage-bearing” (i.e. protocerebral) segment did not express *tio/tsh*, while all other walking-limb bearing segments likely were under the control of *tio/tsh* (Fig. 9) (e.g. [50]).

### Homeotic functions of *spalt* (*sal*)

In the fly *Drosophila*, expression of the trunk-regulator *tsh* is at least by part negatively regulated by another homeotic gene, *spalt* (*sal*) [20, 38], Kühnlein et al. [40], and consequently, in a *sal*-mutant background the domain of *tsh* expression expands towards the anterior and towards the posterior [62]. Because of this negative regulation of *tsh* by *sal*, in wild type embryos the expression of *sal* is abutting the expression of *tsh* anteriorly and posteriorly, but the expression domains do not overlap [40, 62]. In another insect, the beetle *Tribolium*, however, such strict repressive function of *sal* on *tsh* does not appear to be conserved because the expression domains of both genes significantly overlap [6, 68]. Nevertheless, RNAi-mediated knock-down of *sal* in *Tribolium* caused at least mild homeotic transformations like the development of mandibular features in the maxillae, and the development of a tracheal opening posterior to the eighth abdominal segment [6]. The homeotic function of *sal* is thus at least partially conserved in the beetle. The exact function and possible interaction partners of *sal* are unclear. It is, however, likely that *sal* interacts with either the Hox genes directly, or via *tio/tsh*. Knock-down of *sal* in the brine shrimp *Artemia franciscana* has shown that *sal* represses the posterior Hox genes *Ultrabithorax* (*Ubx*), *abdominalA* (*abdA*) and *AbdominalB* (*AbdB*) [13]. In this crustacean species, *sal* is expressed in the posterior segment addition zone from which the posterior segments are generated, and in the form of transverse segmental stripes that straddle the segmental boundaries [13].

Since the expression pattern of *tio/tsh* and *sal* significantly overlap also in *Glomeris*, *Parasteatoda* and *Euperipatoides*, it is unlikely that the interaction of these genes is conserved with respect to the situation in *Drosophila*. It is however possible that the ancestral role of *sal* is the regulation of the posterior Hox genes as demonstrated for *Artemia* [13]. The expression of *sal* in *Tribolium*, *Glomeris* and *Parasteatoda* supports this assumption. The early expression of *Tribolium* and *Glomeris sal* is strikingly similar to the expression of the posterior Hox genes in these species [29, 31] and later this gene is expressed in the SAZ, exactly like in *Artemia*. Similarly, *Parasteatoda sal2* is expressed in the SAZ, and *sal1* is early during development expressed in distinct regions along the AP axis, a pattern that is very much in line with a possible regulatory function on the Hox genes (cf. [67]). Expression of *Euperipatoides sal*, however, is less likely associated with a regulatory function on the Hox genes (cf. [34]). Functional studies will be required to further investigate the potential role of *sal* as a conserved regulator of sequence identity in Arthropoda as a whole, a function that is apparently conserved in at least Pancrustacea. At the moment, the best candidate species for such studies is the spider *Parasteatoda* for which RNAi has been firmly established [49].

## Supplementary Information

**Additional file 1: Figure S1.** Phylogenetic analysis. Tiptop, Teashirt and Tiptop/Teashirt genes form a monophyletic group that is separated from the related Zinc Finger Homeodomain 1 (Zfh1) orthologs of these species. The scale bar represents 0.5 amino acid substitutions per site.

**Additional file 2: Figure S2.** Phylogenetic analysis of Spalt genes showing that Spalt and Spalt-related genes form a monophyletic group that is separated from the related Krüppel (Kr) orthologs of these species. The scale bar represents 0.5 amino acid substitutions per site.

**Additional file 3. **Tio/Tsh sequences.

**Additional file 4. **Sal sequences.

**Additional file 5: Table S1.** Primers.

**Additional file 6: Table S2.** Accession Numbers.

## Data Availability

All data generated or analysed during this study are included in this published article (and its additional information files).
