# Supplementary figures and images for: Panarthropod tiptop/teashirt and spalt orthologs and their potential role as “trunk”-selector genes
Source: EvoDevo. 2021 Jun 2;12:7. doi: 10.1186/s13227-021-00177-y (PMC8173736; doi:10.1186/s13227-021-00177-y)

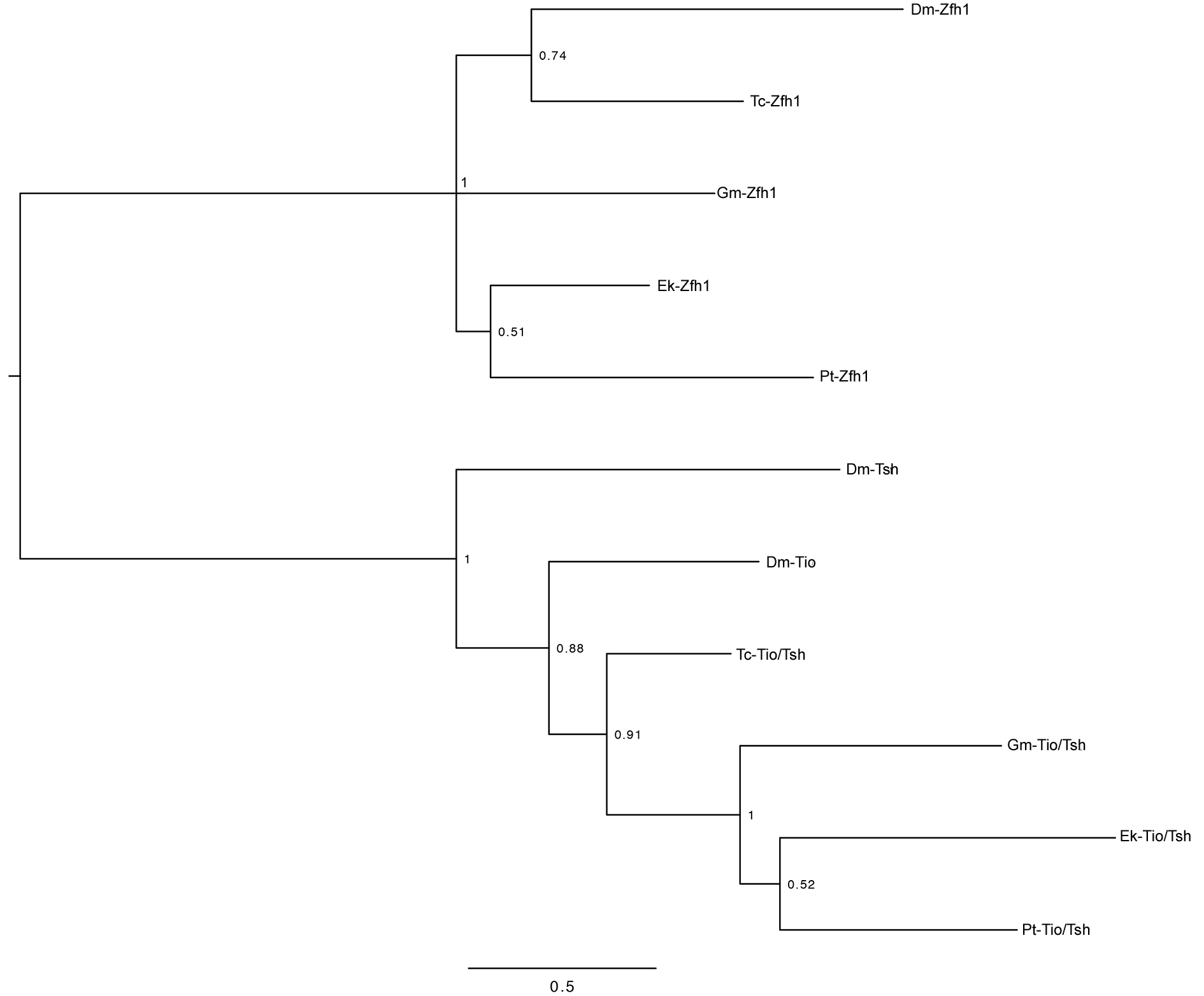

Supplement: Supplementary file 1 — Additional file 1: Figure S1. Phylogenetic analysis. Tiptop, Teashirt and Tiptop/Teashirt genes form a monophyletic group that is separated from the related Zinc Finger Homeodomain 1 (Zfh1) orthologs of these species. The scale bar represents 0.5 amino acid substitutions per site. [file 13227_2021_177_MOESM1_ESM.tif]

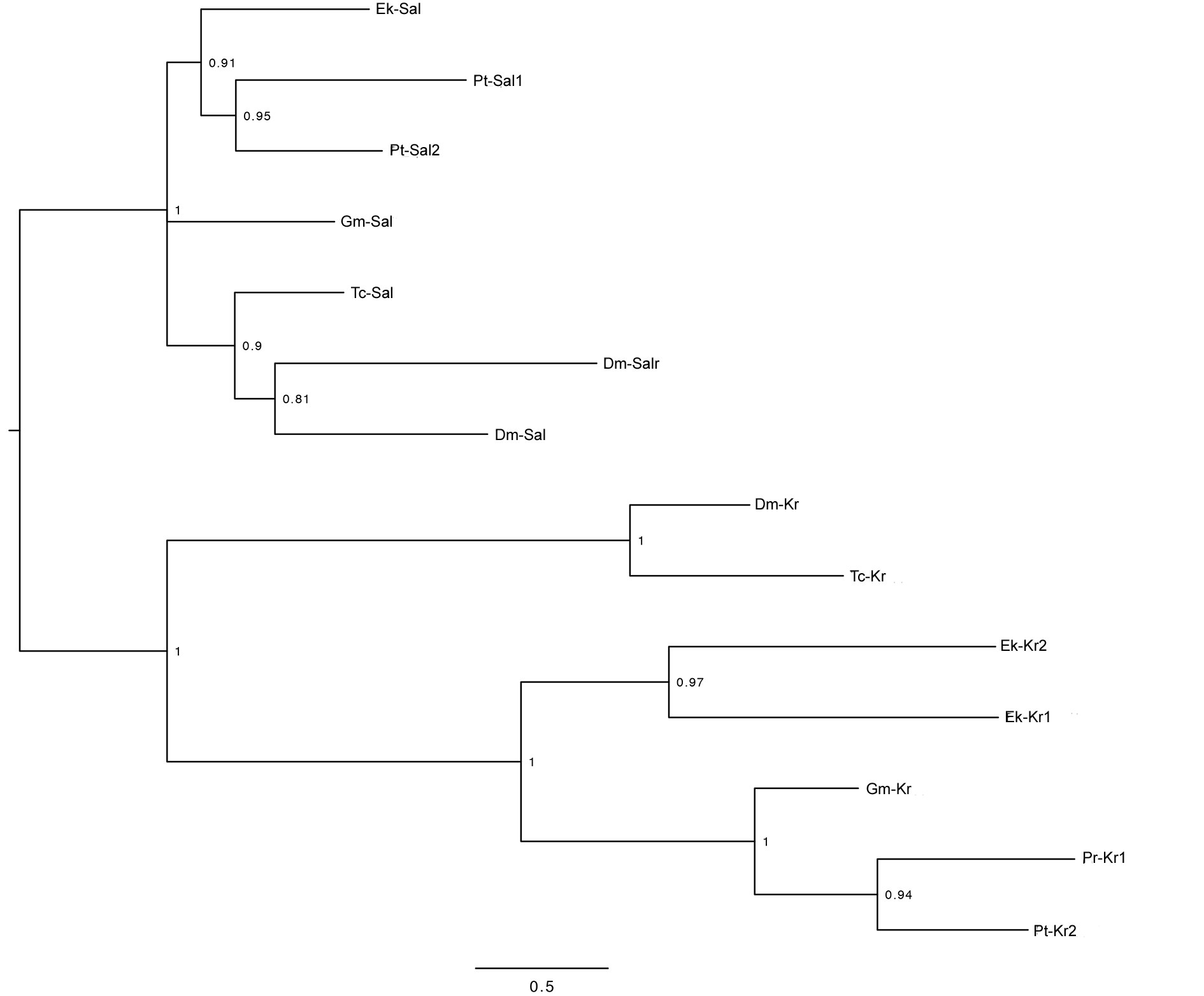

Supplement: Supplementary file 2 — Additional file 2: Figure S2. Phylogenetic analysis of Spalt genes showing that Spalt and Spalt-related genes form a monophyletic group that is separated from the related Krüppel (Kr) orthologs of these species. The scale bar represents 0.5 amino acid substitutions per site. [file 13227_2021_177_MOESM2_ESM.tif]
