# Supplementary material for: Panarthropod tiptop/teashirt and spalt orthologs and their potential role as “trunk”-selector genes
Source: EvoDevo. 2021 Jun 2;12:7. doi: 10.1186/s13227-021-00177-y (PMC8173736; doi:10.1186/s13227-021-00177-y)
Supplement: Supplementary file 3 — Additional file 3. Tio/Tsh sequences. [file 13227_2021_177_MOESM3_ESM.docx]

**Tiptop/Teashirt**

>Dm_Teashirt_AAA28983.1

MLHEALMLEIYRQALNAGALPTARPRSTESANSSERCPSHDSNSSEHGGGAGSGGVGHRLDAAALSTGVMPGEGPTTLHSSFPAVPQSLPSQPPSMEAYLHMVAAAAQQYGFPLAAAAAAGAGPRLPLPLANEAAAPFKLPPQASPTASSNNSEALDFRTNLYGRAESAEPPASEGEEEEFDDGANNPLDLSVGTRKRGHESEPQLGHIQVKKMFKSDSPPANSVASPSASQLLPGVNPYLAAVAAANIFRAGQFPDWNSKNDLVVDPLEKMSDIVKGGASGMGTKEKMHSSKATTPQAASQPPKSPVQPTPNQNSESGGGSGGGAAGSGAVTKARHNIWQSHWQNKGVASSVFRCVWCKQSFPTLEALTTHMKDSKHCGVNVPPFGNLPSNNPQPQHHHPTPPPPPQNHNLRKHSSGSASNHSPSANVKNAFQYRGDPPTPLPRKLVRGQNVWLGKGVEQAMQILKCMRCGESFRSLGEMTKHMQETQHYTNILSQEQSISIKSGNANANSDAKESHNSLSSEESRTLSAVLTCKVCDKAFNSLGDLSNHMAKNNHYAEPLLQSAGARKRPAPKKREKSLPVRKLLEMKGGSGTTQEDHSNEKTSVQGKPGLGPGGGDKNDAALFAERMRQYITGVKAPEEIAKVAAAQLLAKNKSPELVEQKNGGQQRLRASSVLSAIEQMFTTSFDTPPRHASLPASSPSNSSTKNTSPVASSILKRLGIDETVDYNKPLIDTNDPYYQHYRYTSSERSGSECSAEARPRLDAPTPEKQQQGGGHDEESSKPAIKQEREAESKPVKMEIKSEFVDEPNEAEETSKMEAAVVNGSATNNNNNIVERSSPKTPSSAASPQTRLLPPRSPAESQRSVTPKSPASSHKSYDGSSEGTKKFPSDSLNALSSMFDSLGSSGAGANSRAKLAGRSCCWRIRISREPHRRGELLGGLATILREEGEDRLSPRAETKRETESFALFSLFRAKRIFNIFWFVSALPSPRT

>Dm_Tiptop_NP_524733.2

MMLHEAVMLEIYRQALSASELTSPRCQSRDSNTSAGAGAGMADVRCPSNESHCSANDRLTPAATPTLTPTEATISPNSVGLPLTATLPPAAAVALLPPQSAAMAAYLAAAQQNHLLLTNPLAAAASLVQHATQQAVVEGEVESPALDFSRKRPKSHGDDDQEEDQEQDQEQEQEQEPDHDVQCDNGPLDLSVSTGKRQESVSPPARKIPRSISADYKSPLPPGSWMPPINPYLAAVAAKTGGLGYSKLAPSEASKALEKMTEMSRLETSPTAARSLGATSSVGAGVPAGASSNSGGRHSAWQSHWLNKGADTAKDVFKCVWCKQSFSTLANLTAHMKETQHCGVQIPSPLPTGGVGTPSAPPPTRLATSASNSACSSSSSSTSSSSNSSKSELNMLIKETMPLPRKLVRGQDVWLGKGAEQTRQILKCMWCGQSFRSLAEMTSHMQETQHYTNIISQEQIISWKSGDERERPTNTGVPSTSTAAPSSPSCTAPSVSAVLTCKVCDQAFGSLKELSTHMAQKSHYKESPAPSASPPAAGTGNPKRGRQNRNEKRKKSLPVRKLLELERSGSNSSLDSALKPLRDFAAATKITCEKCGSKIETALFVEHIRKCLGESIPIPPRRSNAGVDRLPSPSLGLGAEKPPSVLNALEQLIEKSFESRTSRTMTHGGYSEAGTPLGASILKRLGIEDSSDYTKPLMDAQAMHLLRSSFASRDRSASESSSASRVESSYTPDRQQATPHKSPDTPAPPPPPPPTIKAEPLEAEPLVGCDREGCSPRQQIQVKKEFSMEACRESPRSVSKSPAPQTERSPPDNGSLLALNSMFDQLSGVENSGNNNSGHCFNNNNSCSSVSAQKPKAHPLAALQKLCETTDPPSTGLRSASSAGSSTASATLPSANGNDLVAFSWACNEAVLSASNGGSAGDSSIIKCSYCDTPFASKGAYRHHLSKVHFVKDAGEDSPRLKSPAVQSPRSMPLASPRRSASRSPATGSQQPPPSPTISPYDESPQSKFLKYTELAKQLSSKNA

>Tc_Tio/Tsh_EFA10732.1

MPRRKQDCPKRMKWEGGEEGSPEEGDKLEGDEDTGSMTPNSAASPAPPEEEAASPTPGDVPTPSSPRSISEDPLNCRDLPNSRCNSRESSDSLVQPRCPSGESMLSERAALLRGVPTLSPVGVALPPTLPAAAAAALLPPQTAAMAAYLNAAAVAAAAQQSHRLMMTSPSGFNRASVSPLISSSSSPPGVFPVDAPMHSPPGDGILDFSKRGQKSDADSDSDVVNLSKPGTPPSGEPGNGPLDLSVSSRKRSNDDSVSQPPNRKPRTDFKPQVLPQWPSPIGASHLPYFAAAVAAASNLSPKTNSSPDLWNGKLKHGGPTPSDATKALEKMSELSKLGGEDLFRTMANAAPGNSASNRHSAWQSHWLNKGAEQAKDVLKCVWCKQSFPSLAAMTTHMKEAKHCGVNVPVPPMQSQIINPQPQITSPSNTNTSTSSTNSNKPNSSDLNMLIKETMPLPRKLVRGQDVWLGKGAEQTRQILKCMWCGQSFRSLAEMTSHMQQTQHYTNIISQEQIISWRSSDDAKGGGGGSGSGNNQPPGGPSSHVSAVLTCKVCDQAFSSLKELSNHMVKNSHYKEHIMRSITESGGRRRQTREKRKKSLPVRKLLELERAQHEFKNGDSSSLLDKMRDSSGAGRITCEKCGNKIDTSLFVDHIRQCVGGGTIGSNQRNFLKNALMSNNILPPESPTRDKSKQNEKSPSPQQRSPSVTDLSTKDGTTVPESNNGSSPSVLNAIEKLIEKSFDSRSRQNSSFPGHGPTTAPMGSSILKRLGIDESVDYTKPLVDAQTMNLLRSYHQQQSHHYGRRERSGSESSSISERCSSRVESLTPERKMEPPGVPLNSTPRSTPDKREKSPTSDKAQEADGEVVVKKEVDEEERLENHTSVKIKREAEDRDEDERSFHSNGAKEEDEDKPSVSPLTSPRQPAETHAGSPCRNSASPASSDRSGTPRSTNGDRKPGGSLGALSSMFDSLSGNNTGDALAPSGGKKGSSHPLAALQKLCDKTETHTNNRTHSATSNSSVNANTNNIPTNAGTTPGAILAFSWACNDAVMTS

DSIMKCAFCDTPFISKGAYRHHLSKMHFVKDGVIPDPVALKSAQQGSSSSSEGVPLKGTAVPPPSGSSGPGSTGSKSPPVAGFEESPHSKFLKYTELAKQLSSKYV

>Gm_Tio/Tsh_c59357_g1_i1

MPRRKQDRPKRMKWEDPSGKGAEEDPTDSNGPGSDVDSADSTSRPGSTSPPLDTTSGSQRPPPDDPLDEASMTSDIAGPLALAATPLPPPAPPLLPATPTSRGSAESPPTTAMTPLLNQESPSVAEDQPLDFSVHSKQRRKKRCAEDGGSGGPGDCDGDSDTEEDSCGEDPEMGVAEGDAPLDLSVPRKRSSTSSDHRKSTRNSSSASGTHATNAAPGPWATSGSGPAPFPFRFMTSVSPSSSVELPLWNGHHHHHQQHHHHQKTSSTSPHSSSHSPATTPVHFPTSHNRHTPTPPNPPSRPPSSTPSHPTTPTPPDANLQSKSVEVLLGSKRGCSSSPSNAWQSHWLSRGGAHQAKEVLTCVWCRRSFRSLEELTVHMKEARHCGVGSGSPASCKRSSSSASEEHHHHHQHHSMTSCTTTASGGGGSSSAAKDSVSLPRKLVRGQDVWLGKGAEQTKQILKCMWCGQSFKSLAEMTTHMQQTQHYTNIISQEQIISWKSPEDKASPQAHVNAVLTCKVCDQAFSSLKELSNHMVKNAHYKEHILRSITESGGRRRAAARERRKKALPVRKLLELERAQQGLVSSSSRSVVLDGMGVVADGKVTCEKCTQRIDASSFVEHIRQCLGVQSSKNASAAPDSNHHHHHHHH

>c59357_g3_i2

HHHHHHHQRHHHHHHHGSKSTNGSSSSKTHNSTRNSSKHEGNRLVMEASSASSPLLTTCSSSSSSSSKRKSLLSGSLRVVNANSGDNDPQGPSVLNAIERLIEKSFEYKRGKSVYTSSSTTPLSLKKTSLEDVAAVGTHKVKSDVSDVSGAEDGDDVLDDKTPKPVADKEPVLPASEQLVDGNGDDYHEDTDVGEAEEEPEDKELEADEATAKKRRKRTPKTASSEDRTSVSPASACADAADVPLSVTPPSAAGNSQHCHSPLVTSRPASSGSAVVSADEERGSIVRSPAVDLCTTTTNSGGRSGGLSLVCTTTPPSPSSSDHPLRELQKLLDKTNVSLSSSSSSAKTSTTSTVSIAGGATATVVSGSGSGVGDGGGGSGQDSSAGALLAFRWACSDLLMAESVIKCPFCDTPFISKGAYRHHLSKMHFVKDGTVDGTTTKCPSTAVTTSTTSVVNVSGGGGGGSSSNSSSNSSKPSASPPVLVASSSASSKSPPKTPVDESPHSKFLKYTELAKQLSSKYV*

>Pt_Tio/Tsh_XP_015910633.1_isoformX1

MKLSVKLRPTAEGRSSEILPLVFPPSLLLLPFQREKCYSVGEDGEPGKEGKSSEDHSDTASVDGDDANEQHLDNNSNSSHSSRSSTPELPPPASPLLLPPPPPPPLLKEDREMELLNKASSVMDISRNAVPNPELIQTADEDDDDADEDMASSVATEDETPLDFSVKRSTEDESTDDDRSITPNGLNPPSISQDGPLDLSVPRRRSSRSGGHFEHPRQAKMAKLDSALSVPSSPWGYNAPKHLPKTMVKAAVVEVPMWNGKVKSDKRDKSYHHSVSSSKHSSLNRSSAYNPVMHKNASPPLDSLKVSNKSSKNDPFRPAGRQNPWQSQWMSRSSEQTRDVFTCVWCKEAFRSLAEMTVHMKQSPRCGMAGMQNTLPATSPTSTSSMNHQANLHQSPLPAKCSTSSASSAGPAANAVIKENVSLPRKLVRGQDVWLGKGAEQTRQILKCMWCGQSFKTLADMTTHMRVTQHYTNIISQEQIISWRTPEDKMAAQSQVNAVLTCKVCDQAFGSLKELSYHMVKNSHYKEHILRSITEGGGRRRQTRERRKKSLPVRKLLELERMELNKQNPTSTNDLITQAKNEVVDGKISCEECTEKVDAKDFVQHIKNCAQGSRSQHSVKSSPGSEGAKSMSENNLDTPTTSGAVQSSSPVTSQDLPSEEGSKTPGGQILPQEDSMCDSVESTGSTSVLNAIEKLIEKSFESKGRRNTVSTGILQRLGIDEEVYPPWHPSSSPSAFGALRSSLDFQRKSTSPLGRMHDFKMEMADFSGSESRSRSSSTSEKHPSFDEHLRLTFGLSAKDVMNRVSTPSSASQSCSPVHFQPESPAEDRNAVFLTKEITNSNIASTSQVVSNASPKTRSTTPSSDREIPEKNSAKENEQMEKSKETTEKEVEVKVKEEPEDRASDDEKPMDCSSAISPKSEDRRTPLSRSSSVEPEIALPTSPSAIKSEMSPNKHSKSIPKKKRNHEVHRKSPSSSSKRRSHSHRRKDSDTGNNEGCDSVLGSPLSAHSSDVTSPRQKSHNDSAAGDHPLKELQKLLDKTDAHLSRPNLPA

TPGSILAFSWACSEATTSDSLMKCAFCDTHFISKGAYRHHLSKMHFVKDGSLAEIAAASTWKPPPSSSGNKNSAGASNSNGCSGSGSASGSRTGNNEPGHPASPQKELVAPAPADESPHSKFLKYTELAKQLSSKYV

>Pt_Tio/Tsh_XP_015910649.1_isoformX2

MLLREDGEPGKEGKSSEDHSDTASVDGDDANEQHLDNNSNSSHSSRSSTPELPPPASPLLLPPPPPPPLLKEDREMELLNKASSVMDISRNAVPNPELIQTADEDDDDADEDMASSVATEDETPLDFSVKRSTEDESTDDDRSITPNGLNPPSISQDGPLDLSVPRRRSSRSGGHFEHPRQAKMAKLDSALSVPSSPWGYNAPKHLPKTMVKAAVVEVPMWNGKVKSDKRDKSYHHSVSSSKHSSLNRSSAYNPVMHKNASPPLDSLKVSNKSSKNDPFRPAGRQNPWQSQWMSRSSEQTRDVFTCVWCKEAFRSLAEMTVHMKQSPRCGMAGMQNTLPATSPTSTSSMNHQANLHQSPLPAKCSTSSASSAGPAANAVIKENVSLPRKLVRGQDVWLGKGAEQTRQILKCMWCGQSFKTLADMTTHMRVTQHYTNIISQEQIISWRTPEDKMAAQSQVNAVLTCKVCDQAFGSLKELSYHMVKNSHYKEHILRSITEGGGRRRQTRERRKKSLPVRKLLELERMELNKQNPTSTNDLITQAKNEVVDGKISCEECTEKVDAKDFVQHIKNCAQGSRSQHSVKSSPGSEGAKSMSENNLDTPTTSGAVQSSSPVTSQDLPSEEGSKTPGGQILPQEDSMCDSVESTGSTSVLNAIEKLIEKSFESKGRRNTVSTGILQRLGIDEEVYPPWHPSSSPSAFGALRSSLDFQRKSTSPLGRMHDFKMEMADFSGSESRSRSSSTSEKHPSFDEHLRLTFGLSAKDVMNRVSTPSSASQSCSPVHFQPESPAEDRNAVFLTKEITNSNIASTSQVVSNASPKTRSTTPSSDREIPEKNSAKENEQMEKSKETTEKEVEVKVKEEPEDRASDDEKPMDCSSAISPKSEDRRTPLSRSSSVEPEIALPTSPSAIKSEMSPNKHSKSIPKKKRNHEVHRKSPSSSSKRRSHSHRRKDSDTGNNEGCDSVLGSPLSAHSSDVTSPRQKSHNDSAAGDHPLKELQKLLDKTDAHLSRPNLPATPGSILAFSWACSEATTSDSLMKCAFCDTHFISKGAY

RHHLSKMHFVKDGSLAEIAAASTWKPPPSSSGNKNSAGASNSNGCSGSGSASGSRTGNNEPGHPASPQKELVAPAPADESPHSKFLKYTELAKQLSSKYV

>Ek_Tio/Tsh

MPRRKQDRPKRMKFDEADGEKDDRPLEEETSMEIDDNHEKSTECSSASNSPKLPTVETMKENGDDSGLVQTVNKKWQECSEEPLDFSFKKRTNNNNNEETKDHNCKRPRSTDSSFDENSDLDGPIDLSMPQKRSTKSNDSLTNQNVKSISVIRQTPHYEGLSSKLQASSEYVPVHYGSLWIDETLSNSDSKNLPLKGSQINVSTKHSDILMENINDKDIGKLIKEGIPRKLVRGQDVWLCNGAEQTKQILKCMWCGESFHSLERLTSHMRQTQHYANIISQEQIIAWREAEDKTNPQAHLNAVLTCKVCDQTFSSLKDLSTHMVKLSHYKEHILRSMSDNATKRRYSRDRRRKSLPVRRLLELERGEVTSMDSDSQSLDSDNSELSSSKIRCEKCGEKIDTREFVHHVWKCVGANPNTSNSTQTSAKSNKLQDNPIKTEDNECVKKADKKGKCHKKEKSEKITKMDVNVEKVNIGDRKVEICDKNKSEKHSSDDEKVESCIKKESTESNLIKPLIEKKYENESPPSPENPNGQSVLNAIEQLIEKSFQGREKRTKDHSIKLNHHDFSKPNNSYNDDKKQLFINTESPNLGKSFSISPNNEYSSKSIKVSESSIISGLSQKIDKIVPNGISCNKSLERVTNTPKNGMSESSVLGSYLSGNKEAMDLSLHKIKTEKDIGESVSGEHPLRELQKLLDKTDAHLKQPCTSSTPSLAATPPGYPGAILAFSWACSDALVTNSNMKCAFCNSQFVSKGAFRHHLSKCHFRGRDDISPDKTGWQSSSSSSSTPSKKSPKTELGENPHSKFMKYSQLAKELSGK

**ZINC FINGER HOMEODOMAIN-1**

>DmZFH1_NP_476850.1

MLSCLAPSSSRFGQEDTIIQQSMPSTSPFAMQFPSLASTLLHHNQSPKHSNPGSSGIQDAHPNQPGAAADAFLVKCTQCHKRFPEYQSLSEHIASEHPHDKLNCGAAQPESDAEDEQSNMSGSSRRYAKSPLASNNNSSTANANNNSTSSQSMNNNSELAKNHNSANKMSPMCSPGSLTPGDLFAQLQHPPPQLPPHLHAQFMAAAASLAMQSARTASSPSQQQQQQLQQQQQLQQQQQHQMAMQQLLPPQLPGSNSSVGSNSAYDLDLSAPRSTSSPGSTTGDLSGAYPCMQCTASFASREQLEQHEQLHSPCGPAAVSNVSQTCRICHKAFANVYRLQRHMISHDESALLRKFKCKECDKAFKFKHHLKEHVRIHSGEKPFGCDNCGKRFSHSGSFSSHMTSKKCISMGLKLNNNRALLKRLEKSPGSASSASRRSPSDHGKGKLPEQPSLPGLPHPMSYFASDAQVQGGSAAPAPFPPFHPNYMNAALLAFPHNFMAAAAGLDPRVHPYSIQRLLQLSAAGQQQREEEREEQQKQQQHDEEETPDEPKLVMDIEEPETKEMAPTPEATEAATPIKREESREASPDPESYRSSSQAIKQEQEPLNVAEERQTPVEEHAPVEHAADLRCSRCSKQFNHPTELVQHEKVLCGLIKEELEQHFQQQQATSFALASASEEDEEDEEMDVEEEPRQESGERKVRVRTAINEEQQQQLKQHYSLNARPSRDEFRMIAARLQLDPRVVQVWFQNNRSRERKMQSFQNNQAAGAAPPMPIDSQASLTREDQPLDLSVKRDPLTPKSESSPPYIAPPSGEALNPEAINLSRKFSTSASMSPASISPSSAAALYFGAAPPPSPPNSQLDSTPRSGQAFPGLPPYMLPMSLPMEALFKMRPGGDFASNHALMNSIKLPDYRGTSLSPGGSEKRSWRDDDSRISHEDEFGAGVLMPPKPRRGKAETHGHAGDPDLPYVCDQCDKAFAKQSSLARHKYEHSGQRPYQCIECPKAFKHKHHLTEHKRLHSGEKPFQCSKCLKRFSHSGSYSQHMNHRYSYCK

PYRE

>TcZFH1_>XP_008198751.1

MVARSESMFGCYDSYSYRLWSLANVWNTCQLSPLTNGVSNKESDDLNTTTSSNGDYYIKCPQCQKGCQTFHALKEHMETSHADLTASSPENGLLTTTTTTTSTSPNVAVSPTTPSTGGPFGCSQCTTSFATKDQLDKHELLHSPNAQVSCKICNKTFANVYRLQRHMISHDESAVLRKFKCTECDKAFKFKHHLKEHIRIHSGEKPFECPNCGKRFSHSGSYSSHMTSKKCLVMNLKLGRARPNTPQNHRPLKQQRPINNNINTSPNHNTYLPILPKYSEFLQPPFYLAPPNLPSISPYSIPSLGHIFEQLQQSPLRPPLPPGDDYVQTPPIKEEELKSNTSSCGELVMDEDETTKNEETAPNNTGDLEAVKRILETVNATVTKQLLQANISKFSSESSSSDNSSQHSPKEEQHETSEGLAAKLEDIVQVKEEDEEIDSESVTTTDHVSEDGRKVRVRSLISDEQLKVLKDHYKLNPRPKREDLEKIADTIGFPVRVVQVWFQNTRARDRREGRLIQVPYSPALRYPLVPSSLTISPYNEQPLDLSTKRGPPSIESSTPGSSPRPELDHEAINLTKTPPSPIDFNNSSRLAQILAQPKLSMGTMGLVPMEQWDLPSLSQLITNRLNSLSPKIDDGGDEKRGKVSQYVLKSLGSPVLGGGGDGEVEGQFSCDQCDKAFSKQSSLARHKYEHSGQRPHKCDECPKAFKHKHHLTEHKRLHSGEKPFQCVKCLKRFSHSGSYSQHMNHRYSYCKPYRE

>GmZFH1_c59651_g2_i2

MADQTGSRCTRRKQANPRRKNVEIENMPEEVDLDHLGPEDGMVATEVPTKATSVADEDDDDAAAEDDDELGSVRVEGTTANGDFNPQQNPQSSSVKDEEDEERRRGGGAGAGGAEEEETVEVDSVGGEEVRGSKVNKSSELQLEDDKVNAIRDVDVNVKREEPVTSVPNGFHHRRRRERSKVRAISADDAAMESVEDEDDDDVEDEEDDDEDEERRMEKEEEKIREYLGRSDTAVIYPEPVDADADADADARVNKMNGSSHIACNRQLSALLLKCPHCSAAFSNVQNFRDHLTIEHSAGLAADAALGVDSGLNHSPPPICGNGKVNVTANGNGKESSATVIECQYACNQCSATFQLRDQLEKHELLHSPTTQVACKICNKTFANVYRLQRHMISHDESAVLRKFKCPECEKAFKFKHHLKEHIRIHSGEKPFACPNCGKRFSHSGSYSSHMTSKKCLVMNLKVRRVDKPPRGTSNNIGGAAGAGGVGTGVGVGGGGPASGGPVPACNLASGATRRNGQSSFQLILPKNGANNETEVLDLGIGSRYASAPGLSSSSSAFPVGFPHQGLGQFVPPPYSSLSLHPLLSGNHFPSQPPQPPSAPPALAAQQHLPPSSSPSRYSLPSSVLQIPSGGKTIKDENEDQDSSEEGRFSCRKDEDMDIAGKTANGDLNAVQKILKAVNATVSRQLQEVTTNNGKMKNGSGNELSDVKSEPRNGTGHEDGDNICCVICHERFKTYLDIALHRQKGKCSTNYEDDEDAELNGDKVKASEAMEMSCNLCHAVFQSKIDLHQHERYLCPRNHEVLRVKSNSSNDDLKVEVKRERLSEDHNSDEDVTRKNRDVAAVSSSMDHDNSGEECKEDTLKSRLGKLANVHHYHHQPSMTVSETQVQLLRAFFARNPRPRKPELGKIADEIGLSLSMVQAWFQCARFHDRKGIHRPSTYDNGLPPSQYNSTNSTPVYPHMVSPFNGVIPLRISPTYYPSPTSHHGSSSTLPPPSVPIPPQSHHQIQIPAFLSPITGDRLNGQHVPSSSPPSASSSSSSLNPHFQPLNLSRESVIEGSPLGGVGGGGVIADREGATDAEQPLDLSVKSCRPSPARSSSPTNSDGEALNLSRKSSRTSTPYKESNNAEISSSNHQQQHHHHHHRHHLTVAGSSSPFSTVEAIRTTPNMVSALTGTPPPPLRGPGSVTSSSPSPSPSSNIFLPHGLAHHHHHHLHNHNHHHHHGNHIGASQHIVLTGHERPASDTASSSGVMYRGDVDDDNDVARVSSYGWDADDGDDTLEVDESASEGKKKIWKQLEGDESQAEMNDSSLGEDDRPLKRRRSWKQHKVDAEEGQRPHKCDICTKAFKHKHHLTEHKRLHSGEKPFQCKKCLKRFSHSGSYSQHMNHRYSYCKPYRE

>PtZFH1_>XP_021003029.1

MFYNSMWGNCGGFGRSFGPDIMWPAYFNRFLPVDLEGMPEDADADQHVAMRPSLTSPPLNTPLPAKGPLLSTSSKRAQTEDLPLTNGTSDDRPSQINGISKKNPEDEKIQEYLQRSDTAVIYPEPVGGMSPKQREDKHQSGSPEPVPVTEESSLKCPRCDKIFSGQQAMRDLKEHHSVHHKDADPPESLFTCQKCNASFTTKENLSKHKVYHVSNGQSSNVQTNDAERRFKCSECGKAFKLKHHLQEHLRIHNGEKPFMCPNCGRRFSHSGSFSAHNTSKKCLVVNLKVRKVDSRSPRGRGNSQNNSLRPIIPKYRSTGAGGSITSSAPTEMPLIPAGYMSSPERFSSFSSRPQQPYLPLTFPHPMHYLPMSTFPEVTQLLQQRRYENEVENHSQLPPQILPTSSHLDKSDSSPRSSPKPQGELNAVKKILEIVDNTVSKQQQSPTRPRNGLLSELLSAAPQSSIKSPPPYNDNRCRYCSRLFDSNIELHQHERYLCSSNIELRSRPESPRENGASETESCEDSNRVCMSPSALSVDCELTLKAHFQMNPRPKKSELIRLAHDLKCSVRTIQEWLQRQQMRSKDYAMNGNASPSPTPSRQQAISFTPSVLSDCNGHPFVPHYNGAISSCAPLVPFRPIPESPRLNVEEDQPLDLSFKFKKEDTDLYPPEKSQSPVNHLESEVLNLSQRSSRTPPKVDSPSSVQSNRLSQFPGQNNQGEDSSAQTTSLSSLHSSLLYKYMQMGMSGQQQQPREDRIKSPMVEAVLSPDHSPSRADQPLSMSSPGGYANNSRSPNATTGSYYHAEEPTDSSLYSPSAKKPRLWNQGDGEDSAMGDSPGPEEECSVGSSGKARKSWKLHKVEAEEGMYACDLCDKMFSKQSSLARHKYEHSGQRPHKCDICGKAFKHKHHLTEHKRLHSGEKPFQCSKCFKRFSHSGSYSQHMNHRFSYCKPYHE

>Ek_ZFH1-like_c209198_g4_i1

MPDEEVLIHLGSKDTLTSPNHSEPVEEDHEEERDNCLSETLLNHSNPSSPKNNPEMASTCLQNGAPSPSSTNKPDEKKLESSMLGRVALENGHQKKLDGLEKLMDDKSEEEKIQEYLRRSDTAVIYPEPVEGELDRPPSGSTVHDSTEKVEGEPDLRCPYCEKSFARLLQLREHMTMTHREHEGNHSCPKCNASFLNRSHLDKHLALHSPNSQICKICNKTFANVYRLQRHMISHDESAVLRKFKCPECEKAFKFKHHLKEHIRIHSGEKPFACPNCGKRFSHSGSYSSHMTSKKCLVMNLKVRRAEKPRVRRNGEQGLRPIAPKFDSVGEMDLSVSSYPEQCLEFPRHFVPPFASTPINTLLTNHFQTAGLCSAFRPLPALLDSAGYRIKDEAVEKHEIQQSISLSMALDKDSSSNGSHRNELLTTSIPDVVVKKEEEEGVNKGNKDLNAVKKILAIVDATISKRQQEANMAKLKNGLIQEVIQAHSRPKQDSSSADEASNSSSAKADSSRTERSSCRFCRENFESRIDLHQHERYLCKLNKEILQVAVMNNGDVIREAASFKLGQNGLQGSDLDSDDNDSNKDFVDDEVYTSDGKKMRVRSLINEEHLRILKAHYEINPRPKKYELVRIGREIGFPKRVVQVWFQNMRARDRKKGRAIHVPYFPPGDNSPITNLRPYSLPPNINPSPFSPVLSPFNGGVPTHHTLPVSLFYAAPHSTRFAHSNLAISPPYVTSPYTPPPLLALPAPEEDQPLDLSLKSKERGLVNNNIIIRYPRLYASSHLQDCDESDYTVLNLSQKSRASSPTESNNNTKTHSMSHSNNTDNKLFKSEAKLEHSVLYKYMQKEGLIINERTRENLARKDMVETNVDSTVHDLNFCTINNNEPRSCVELVLKSEPSAFSPLPRTSTPDKAQLSSHVTIEEITTSSGDNSHGPYDSDLTNTSQDPIDIEKIGLKIKRARKKSWKQDSKMVESEEFQYDLDETSSVDGEKPAKHRKSWKKHKVDIDEGMYACDQCDKMFSKQSSLARHKYEHSGQRPHKCDVCGKAFKHKHHLTEHKRLHSGEKPFQCKKCLKRFSHSGSYSQHMNHRYSYCKPYRE
