# Supplementary material for: Panarthropod tiptop/teashirt and spalt orthologs and their potential role as “trunk”-selector genes
Source: EvoDevo. 2021 Jun 2;12:7. doi: 10.1186/s13227-021-00177-y (PMC8173736; doi:10.1186/s13227-021-00177-y)
Supplement: Supplementary file 4 — Additional file 4. Sal sequences. [file 13227_2021_177_MOESM4_ESM.docx]

**SPALT**

(ZF-x_7_-ZF-X_11_-ZF)

>Dm_Spalt_NP_723670.2

MKNHLSNVLCAMRSDFKDNHQETINKMIQFGTVKYGIVKQLKDRARSADKDIGSDQEENGGCSPLTTATTTASPSRSPEPEEEQPEEQSTSEQSIPEQSTPDHQLENDIKSEAKSEIEPVEDNNNRVAMTKPSSEEREPNASGSMPSSPVAEASAEEAATERTPEKEKEKDVEVDVEKPDEAPSSAVPSTEVTLPGGAGAPVTLEAIQNMQMAIAQFAAKTIANGSNGADNEAAMKQLAFLQQTLFNLQQQQLFQIQLIQQLQSQLALNQAKQEEDTEEDADQEQDQEQETDTYEEEERIADMELRQKAEARMAEAKARQHLINAGVPLRESSGSPAESLKRRREHDHESQPNRRPSLDNTHKADTAQDALAKLKEMENTPLPFGSDLASSIITNHDDLPEPNSLDLLQKRAQEVLDSASQGILANSMADDFAFGEKSGEGKGRNEPFFKHRCRYCGKVFGSDSALQIHIRSHTGERPFKCNVCGSRFTTKGNLKVHFQRHAQKFPHVPMNATPIPEHMDKFHPPLLDQMSPTDSSPNHSPAPPPLGSAPASFPPAFPGLQNLYRPPMEILKSLGAAAPHQYFPQELPTDLRKPSPQLDEDEPQVKNEPVEEKDQREEHEQEMAECSEPEPEPLPLEVRIKEERVEEQEQVKQEDHRIEPRRTPSPSSEHRSPHHHRHSHMGYPPVVQPIQPAALMHPQSSPGSQSHLDHLPTPGQLPPREDFFAERFPLNFTTAKMLSPEHHSPVRSPAGGALPPGVPPPPHHHPHHMARSPFFNPIKHEMAALLPRPHSNDNSWENFIEVSNTCETMKLKELMKNKKISDPNQCVVCDRVLSCKSALQMHYRTHTGERPFKCRICGRAFTTKGNLKTHMAVHKIRPPMRNFHQCPVCHKKYSNALVLQQHIRLHTGEPTDLTPEQIQAAEIRDPPPSMMPGHFMNPFAAAAFHFGALPGGPGGPPGPNHGAHNGALGSESSQGDMDDNMDCGEDYDDDVSSEHLSNSNLEQEGDRSRSGDDFKSLLFEQKLRIDATGVVNTNPVRPRSSASSHGHSVG

STSAPTSPSVHASSQVIKRSSSPARSEASQGALDLTPRAAPTSSSSSRSPLPKEKPVSPPSLPRSPSGSSHASANILTSPLPPTVGIDCLPPGLQHHLQQQHQHLMQQQAAVAAAAAAQHHHHQQMAALHQHQEQLRREAAEAQQKAAAAAAAAAAAAAAQRQTPPQARDQRQEGGPGAGPPPNPLMGARPPFGMFPNLPLFPPATTQNMCNAMNQIAQSVMPAAPFNPLALSGVRGSTTCGICYKTFPCHSALEIHYRSHTKERPFKCSICDRGFTTKGNLKQHMLTHKIRDMEQETFRNRAVKYMSEWNEDRE

>Dm_Spalt_related_NP_523548.1

MCSIFRNRINYRGTGGTRSGSGERERDRDRDRDRDRDRERDRDTLLAKELDADSNNNGTEPQMEAEAVPESDTERETAEERGEEQEPENSNEALDLSLISSGGRESLPGSGHVSLEALQHTKVAVAQFAATAMAGNHQSADLAMVQSTIFNVQRQHLMQLQLIQHLQSQLKRAEAAALGRHSHSDEEEEEPEPEPEPKKQPTNGLKEELELEQGPESEQDQESRREENSKTDKRGTEDRKAEPEGYQSMMCDISSSLASSIITNHDPPPAPNEPNCLEMLQRRTEEVLDSASQSLHAAQMQEEYSEYASKEAQSRGEIFKHRCKYCGKIFGSYSALQIHLRSHTGERPFVCNVCGSKFTTKGNLKVHYQRHTQIFPPMLLPPGVAPNVGHSGQGQVQGEQYPIRLPFAPPVAPVGQEQHQNQVEEPEEIRQEIPVPQAEDLSKPMVKEKEKSHSPVERVKTPKEVKTDAALPSSEKPEKEISKPVVTSSRRNGSVRKRQTSAVSPPQEDRERDLVEHLHIAKLVRRSSASRESQPAEYSLAQMERIIDKSWEDLIEIDKTSETSKLQQLVDNIENKLTDPNQCIFCQKVMSCRSSLQMHIRTHTGERPFRCKICGRAFATKGNLKAHMSIHKIKPPMRSQFKCPVCHQKFSNGIILQQHIRIHTMDDGSGGQGAPAANPGEAERLGIEDQNSNKSLGTSDTLDFSTTISDHSGQRSESSQGGDFDEFMTMDSTDDSRDNSSAATATPHPLERERDREKERERRIPNDCSDERSHSNPDLTGGRSESGEMPAMDLSSPSSNSGRIFATGLANGAAGGGSGNGGLPMLGMPMPPNLLLMAAAREEMHALGHAHAKFPLLPFGPLGFMGLHPPPNVCNLCFKMLPSLAALESHLQSEHAKEPATGHAQRPQCSDAGSPYGAKLTLNPNLFAKKPPSSSSSSSGEKLPESSNPPFPAENPPATPIKEDPDQEQLMVEEGASAGEGSGTGATSNYPQEAGDAEQSLMKMQLHAHRFPASPLDFQQALMSAGPPTSSLDPPVNNKHFCHVCRRNFS

SSSALQIHMRTHTGDKPFQCNVCQKAFTTKGNLKVHMGTHMWTNPTSRRGRRMSLELPMRPGPNSGQGHPGSSAEQEFMQRRPELFFPYLPPFFNGLPPKPGELSPGAFPNIPPPPFANGGKYPYPPGLLGFPGFLAQHPYILERRSSSKSPTPEPAQSPALREEEGSGNIWHPLSRIKVENNQNESMGGFNEQEDHAETDATGGDNDESESRDAEK

>Tc_Spalt_XP_008193710.1

MSRRKQARPSRLLEDDDAPAFPAAAGLDEASPCMDRDMSLTSSQDLPEVLMCQRCQEQFTDVEEYLTHKAECEKKAIKHDDPAHSDPEDMVVSEEDDEDDDAGGKRLERMRRHRQDAANNNSVEENSPEDTSPRLSFPIPIPSAPGHVTLEALQNTKVAVAQFAATAMANNTDNEAALQELAVLQSTLFTLQHQQVLQLQLINQLQQQLQIDRSKAASPVSPPPSENGENAPAEASPPPQNLPVSREPTPTPIIQPPQPIVQEPSENQTTEMNVPCSLPLQSQHCSISSSLASTIITHNNEPPSLDEPNTLEMLQKRAQEVLDNASQGLLATNLADELAFRRNKGSLSPYDSKGRNEPFFKHRCRYCGKVFGSDSALQIHIRSHTGERPYKCNVCGSRFTTKGNLKVHFQRHSAKFPHIKMNPNPVPEHLDKYHPPLLAQLGQQPSLSPGGPPPHMGFPGGHPFPPTSLPLYRPHGPPPDLLNSRLQPSPHRPQDPPQRLFPPHPLFMKREEQEAPENLTKPARSPTPVRDTHCKSEISDEKREYDDAQSNVPQITPKQEPNDEGEHEPERYSSPAPYDECSIDSKYSNEDTLGARSPGGDHSENMQDEPENLSNKSNSITVPLSISTGQRLPANFSFGQVNSPPSSTSSGSLGQFPATPVIDPAKDPAIYSNLLPRPGSNDNSWESLIEVTKTSETSKLQQLVDNIEHKLSDPNQCVICHRVLSCKSALQMHYRTHTGERPFKCKICGRAFTTKGNLKTHMGVHRAKPPMRVLHQCPVCHKKFTNALVLQQHIRLHTGEPTDLTPEQIQAAEVKDFPSPGGFPSIHNSINPFLGQGFAVPGLSPLGHTLSMNHKYEKIDKEEHESMDDDDDDDDDMSNSENQNPRFTSSPDMQGVPTSMASQLSMSGLSCSAEDLCSTRTAASASPMQNGEKSPCQSGITSPGSSEIRASPNRVTTTPVQVPRPPSSQHAGSPTPSECNSLGALDLTPRTNQPLITSPGPSPGPPPALFSTFGLIPPGQGSTPLMSSALSSLTSSVLTSTAFSPLRLAVGPTGRGNTTCNLCFKTFACNSALEIHYRSHTKERPFKCSICDRGFSTKDGCVCRERRIQDEAEPWKASKRKPASRKSIPSVLPLPMSPGYASNWMHIAGNMKQHMLTHKIRDMPQHMFENKPPISGDENSQQSQQNLPQREVQPSENEQPNPPSQPSISEQNKEQPVKREPTETELPLPKRPPSLQASKHLCHVCNKNFSSSSALQIHMRTHTGDKPFRCTVCQKAFTTKGNLKVHMGTHMWSNGASRRGRRMSLDLPPIPMTPKDSEFLQRRPDLFYPYLPAPFLNGMQQKLNEMSVIQNNQNGMSAAGKYSGLLGFGYPPPEALRSQGGSPDRPERPPSRDPESRSLWDLHFERKSAPEAPREELLPASREGLAA

>Af_Spalt_AJ567454.1

PAKDPAIYTNLLPRPGSNDNAWETLIEVTKASDTTKLQQLVDSIENKMSDPNQCVICHRVLSCKSALQMHYRTHTGERPFKCKICGRAFTTKGNLKTHMGVHRAKPPSRLLHICPVCHRKFTNGIVLQQHIRLHTGEPTD

>Gm_Spalt

MSRRKQQRPQHIGSEQDLATLLQNGGCGGGVEGVSSDEEDTGDEHICGRCRAEFVELADFLRHKKCCSRKRVVVIVADDATSLSDEDDTHMMTADDEDYHHHHHQLSVTTGTTTTASSNHPLSQGAFPFLLLADPKGGVGPAPAHQNSPGSSSDLVVLQSTLYSLQQQQLLQLQIIQQLQQQLVASGNPPTGALAVLAAATTPKTASAAVAAPPTSSSLSAAAASTVAASGSGSVVASGPSASARTTSGSAGGGAAGAGFQQPQQQHHHQSVSGHVVGGSGGGGVASSQISSGSSPLTIATAPDGHDALNQPNSLELLQRSAQQVLNSATQGLLADLHFRKSGSKSGGGGGGGGGGSRSSAGRDSEEPFFKHRCRYCGKVFGSDSALQIHIRSHTGERPFKCNVCGNRFSTKGNLKVHFQRHKAKYPHIRMNPHPIPEHLDKYHPPLLAQGAQSPPLPPPSRPHHHHHHHQSQHHHHHQQHHHQMQHNAPAHLHPHALPLLVPSSHHHHHNHHPLHHHHHHHHRQQSGGGTVGPTTPGDQSSLDPAVYAPLLPKAGSNDNSWESLIEITKTSETSKLQQLVDNIEHKLSDPNQCIICHRVLSCKSALQMHYRTHTGERPFKCKICGRAFTTKGNLKTHMGVHRAKPPIRVLHQCPVCHKKFTNALVLQQHIRMHTGEPMDMSPEQIQAAEVRDFPGGGPPRQHHPFPPPPPPHHLHGHHQHHHHHLQTSRHSTVLKGPLPSPKMESSANIDDNVTSGVIRSGSSSSVPAGGDVAVADNADPVAATTNTNPVPSPSPTSLLPPPPPPPPPPPLPPPTTTSFSASLAALENHVKAIHAPHLSLPHFGGGGSGGHSTGVLYGERSSPDDRSGDGTNTTASVKSEGSSPVHHRHYPYGVATPVLVSPSAGGGDSALDLTPKSSSVITTTASSRDGHQPASLSGGPLGGTLAGLGLEGLTAALGPHQLPLPFPLTPPGRSNTTCNICYKTFACNSALEIHYRSHTKERPFKCTVCDRAFSTKGNMKQHMLTHKIRDLPANLFEQTTSTSQSLLQTTTTTTTSASPATSPMTTATTAPVRCQQTSSPPAAVAAVATTAASTPSSASPTSSTSPSLTPGTRGPGAPPAGDTVEAGGGERQHHSSHGHHGSSGGGGGGGGGVKRSPGLPKHLCHVCNKNFSSASALQIHMRTHTGDKPFKCSICERAFTTKGNLKVHMGTHMWNNGSSRRGRRMSIDLPPLPLTPKDNDFLQRRPDLYFPYMTSPFLNGMAAKMNEISVIQTVMNNGGLQHHSVPRPSRPPPPPNQPSSQVVPVPLKSTIPATTATATTTATTTTTTTSSSSSTTTNNNNGGCADHFTDNEKSPPDITSPMSQSPPPVTSPMTETPPSSGGGGSSQWLWKLSCSICGRECASTSELEVHLKGHYQPTTDTEAPPPGPKLLENVSSSA

>Pt_Spalt1_XP_015922317.1

MARRKQRKPKHLENAEELATSLQNGIGSDSETEGDEHVCGKCRAEFPTLSDFLLHKKICASKRPVLVAPLDTTMETDDEENSDDMEDGFTPPPPQLEGAQVDSPQVAEWLLENNKNFRSLLNTQMPCTNVTLQGLENTSVAVAQHSQLEEMLIQGLFNVITRNQQESMKLFKEWAKQFLASHPLNVAPLSGAPTVTSPQSTENGDHLESNAPPSNPLITEPYSIAGRLQQNLSMTNLSLDGPPETTNSLALLEKQADRYIQDSMARRSFLLNGIEDDNRSKKGKDDPATRHKCHFCGKLLGSDSALQIHVRSHTGEKPFQCNVCGSAFSTRGNLKVHFQRHKERMEIPGSFLNADQKQSDSQPSMPPESNSDNPPMLYGNDEDNHRGDSSKIEQALDFSTPLNGRKSSNPDLNSGSLSPNDTSNFSDNSGDGSSDDNMPDDYSDLKIDVERFDDEDRDTSNLDDDGSEVASHPSFGSPNFAPYFLSPYDYPVSTATSTAGNTTVSNSITNTKGANGDNSEAILQDPSYYQDLLPRIGSNDNSWESLIEITKTSETSKLQQLVDNIEHKLVEPNQCIICQRVLSCKSALQMHYRTHTGERPFRCKLCNRSFTTKGNLKTHMGVHRQKPVARLMHQCPVCHSQFPNAIVLQQHIRTHTTDHVMQLPPVFHPIDDRPPTLLPPSYPRQFLLQSQLVNSEPLALTSSKSQEDSKGSPANESVIKVTGLENNHVNTASSPLSARSSSPSSNMEESFEEKESKPPILNLAQSDRDVSPQKEVKPDSETYEVTLKTVSRNSTPEKDKITDSIPMDLKSNIKSSPKQESDQTPSNNEDSERESTDTPNRLEKLSPKFVSPPNTSLLVTLASDRPLPMTLPSDCPIPFSVHGPDRHYPPMSISNCSDYSTASLAALENHVKGINSSVTHPLPFAPHFGLGINMAGYFRDYNYPQKPTASPTINKNNTDPSSEERTTPGVVVTNGKGETPRTSSLSNQTLSKHDSNNNGGGALDLTPRSSPAFQRPATSDLKIFPPLAGLPFGPPHHRSGTTCRVCYKTFACNSALEIHYRSHTKERPFRCEVCDRGFSTKGNMRQHMLTHKIRDFPPQAFSTNSNSNSSPITSSPQKETSGEKSPKKVDEGVKRQSTDVALPPVPAKRAPGTPKHLCEVCHKPFSSGSALQIHMRTHTGDKPFQCHICHRAFTTKGNLKVHMGTHMWNNSTSRRGRRMSLDLGPLQLNPGAKPGEFAQPPYFPYLNPFMNGLPNHPHPPPPPPKMNEISVIQTAGMSNGSSASAPITTSPSVTTTPQPLSLLSSNKQLDRSSENSHRQNQIQSHSQGKDIHPILQSSQEIPASWPWKISCNVCNKICSSSTELELHIRNNHCNKKESAETERTD

>Pt_Spalt2_XP_015918429

MSRRKQLHPLHLDAHDQLGSLLDNGSCNEDEEAGLGDEHVCGKCRLEFIDLSDFLHHKRVCTKKRLVLVGEDDSSDEMGAEDEMEISSMEFKEETEKYEKAIHTIPYFMAFPRVKNCFEETPVMPPSTIESKKDFSEGEEQSIDLEHYPKTDIYENNSDDSNQPDLKKCATEANSFSNPPPLPQYLLMHSVPDTNVTLQALQNTRVAVAQQGAGTANLSHLALHTALYTLQQQQIIQLHQVIQQLQNQLVNSSTVTNPLITTTPTIGSLTGSAALTPPQSTDGNNGGILTLTTTTPSTRAIESVTQAENHTVAVSTTPSLTTCRNSTSGSTTPIVSEPPPPPPHEPNTLELLQRHTEQALQNTMAGSSFLLNGLSGIGSSSDILRFCKKGDIKKEGGEDAYFRHRCRFCGKVFGSDSALQIHIRSHTGERPFKCNVCGNRFSTKGNLKVHFGRHKEKYPHIKMNPHPVPLHLDNLHPPLEPPEASETPPIITQTSTCQTVALSLQPPNLVPAQPLPLVTHSHEKTLLKEKESSPSDTIRHSESSFQIDRRSVSPNGTSNLSDDSDGSVGDYCTDENIMKDGDQNHYDDDMDNDDDDSFSDSPKTEQKELDDNMDDEAKTPTTESNGPLCLTSSIPSYTSNAVPPTFPYFFHPSRSPPSSISNPNYPPMSTPPLTSTPSNRISSVSHTNNDGNEQVGPSHDPNLYQDLLPKPGSNDNSWESLMEITKTSETSKLQQLVDNIEHKLSDPNQCVFCHRVLSCKSALQMHYRTHTGERPFKCKICSRAFTTKGNLKTHMSVHRAKSPLRVLHQCPVCHKQFTNSLVLQQHIRMHTGEPTDMHPEQIMANEVRHGHLLTTSFPRPMIPTLLPTFPTPTSLAPRASSAGPPNICTTPKSTEATSLSPSPVHSSSIHSESVIKTTVKQADNLTTENSSSPTTIGNSSRPSSARDERSETPQPPVLISHSSPKESSTPQMSPPSMDKPIVTTAPYSPAYTASLVALENHVKAINTTIPQPLPFSSFGMGLAMGSFLRYEDAGYLNRSSHMNDSNCEKSLINHRSYSPSPNTSRAGSDASGDERSTPGSIIKSESPVSMNNNHARIATGVITPKSESGALDLTPKNSLMSIPQSVADIQLFPSPLAGLPFPTTPGRLSTTCRICFKTFACNSALEIHYRSHTKERPFKCNVCDRGFTTKGNMKQHLLTHKSRDLQPLLFASADNSVQESETNSSTNATSPRTSSPMNAYEANLSQSSNPPEEKKDIPLITTTSNPSMVTPSPVTAIISTVSQIAPSIVSTPLQKTIKTESEFNVKRQISENNSSSPSKRNSGFPKHFCHICHKPFSSASALQIHNRTHSGEKPFKCVVCGRAFTTKGNLKVHMNTHMAQNNGNSRRGRRMSLEFQPPPLPAHGKPDFPPHARPELYFPYFPPGYINGMSVPPKMNEISVIQTAAGVTNGNISTSTAMSLTPAVSMMTPVMTLGQQPFIRKQVQQNSDGDNGDYADPRSPDEAENLKIQPSPSPPLESASPVRPVPTSPSRASNNASCWSWKTACNICSKICSSSSDLEIHLKSHCNPAIQDTVRSASQSAKNLAS

>Ek_Spalt_N_incl

SSLNQSSPLTPQPSLDTRSSHIMPGTPDIVSSPLNPCSVPNHGSPRIDLNNSEHSSPHVIETPTSVSTITTSGSAPPIPLPPTNNSVISTSAIVSPTQAENYFTSLLPKTGNLDNSWESLMEVTKTSETSKLQQLVDNIEHKLTDPNQCVICQRVLSCKSALQMHYRTHTGERPFKCKICGRAFTTKGNLRTHMGVHRSKPPMRLLHQCNVCHKKFTNALVLQQHIKLHTGEPTEMNPEQIQAAEVNDYPHSEMAASGYSSSGESSNTNVIKTEMKPDVAKISVVPNVPTTTTTIVSTFSTSLAALENHVKGIYTSVAQPISFAGRSIDQMCRPLIKTDTPRSQSPVHNRNGDRSPRMSSPPLSCTPSESSDGGPNSPGSKPEEPVNMSTSSMVSGSDGSNCAMDVDNGALDLTPRPMSAEGHALTSSRNTTCNICLKTFACYSALSIHYRSHTKERPFKCNVCDRGFSTKGNMRQHMLTHKIRDLPSQIFESTPKPELPALMFRTEIIPPADSGKPPMPEGGDPSMQIPRRPSSSKHVCRVCTKPFSSSSALQIHMRTHTGDKPFKCTICDRAFTTKGNLKVHMGTHMWNNGSSRRGRRMSIESPLTLSHNGNEFLDGLRHSTEMYYPYASPAFMNGLAPKVNEISVIQSPPMNNGMFPPISLASSETMSLLKNNNDLELNLKKSPPPSSTSMSPVPPLTQAPSQPVDANKNASWVWKTTCNVCHKVCSSASALEDHIKVHVQGEGEKLTA

**KRÜPPEL**

>Dm_Krüppel_NP_477467.1

MVYYSANQLLIKTEQSSQAQFCLQVPPPLTATTTSVGLGVPPSGGQQEHFELLQTPQQRQMQLQLQDQHQQEQQQFVSYQLAIQQHQKQQQQQQHESITNAAPTAAPSAQRIKTEPVGGFPASAAVVSQVRKPSASKPQFKCDQCGMTFGSKSAHTSHTKSHSKNQDLSLNGASGAGVAAPVSTAAIELNDAGLPVGIPKSPTIKPLANVAAGADPYQCNVCQKTFAVPARLIRHYRTHTGERPFECEFCHKLFSVKENLQVHRRIHTKERPYKCDVCGRAFEHSGKLHRHMRIHTGERPHKCSVCEKTFIQSGQLVIHMRTHTGEKPYKCPEPGCGKGFTCSKQLKVHSRTHTGEKPYHCDICFRDFGYNHVLKLHRVQHYGSKCYKCTICDETFKNKKEMEAHIKGHANEVPDDEAEAAAASAAASTSAGSSAGSPSLQGVSSNSESSNHSPPSSPPATKKPRQARQPRVSKTVAATLSIPTSSPLSPSSLSSTYSPSASSMASPPPTSAHYLPVQMEADALSRDSGVSSAQPAHSTYADEEPTDLSMQQVQGQLPESTVDYYQAPPSLLELQPQPAGLTINPALLEAASIARRHDDNDDQVQDEDVHAAAWQMMQLCRGHGSLPPTEQPAPSHQPQVPTLHVSDLAANYDDTHEATVLIEHFKRGDLARHGLHKGYAPVPKYESALPNPDVVRRVEAAIGLRSSTESPERSSSPESDSLMMADRNVMTLPLRKRKHYMNKGDDGQVDSEKASGDGTSAAGGAASVGAGDGPGSKVMRMSSVIQFAKAS

>Tc_Krüppel_XP_008194043.1

MAQKSADGGDKVQEVFIKNLFMRCKEERSETPWQMPEMVGYYTEDPLAIAPVPTVDEARLSVKKVVCSPDLPMPEFPSGAAPHPDQTINIQCQICNKMFATKSAFQAHQRTHTRETEDPYRCNICSKTFAVPARLTRHYRTHTGEKPFRCEFCNKRFSVKENLSVHRRIHTKERPYKCDVCSRAFEHSGKLHRHMRIHTGERPHKCDVCSKTFIQSGQLVIHKRTHTGEKPYVCTVCSKGFTCSKQLKVHSRTHTGEKPYSCEICGKSFGYNHVLKLHQVAHYGEKVYKCTICNDTFTSKKSMEAHIKSHSENAPTPPASSTSNESSCSSSTSDKENKDSLPMPQDPLSYDSDIRYLIYPRDSGYMVPQTGVELLAAAATATEREDVVFNIITKNPVMIRQPYFQTPSVQYTPLNAGDAIRKKVEAVLGVGEMPSSEEENILTPPSSNPVSPAASPVSMSSPDRELSLPPRKRSRMILKSLEETIDLSPVRYSSVIQYAGAS

>Gm_Krüppel

MKEGSEGDAVPLVLSKKPNPGSAASSPGACSPPTSSNPTAGASPTSYLSSSRVQLVGASLVSLGGDVAGAPCAAGPSAKVEGNHGRRSLQEDAVAAAVAGAAGGPRDKIFVCSVCNRCFGYKHVLQNHERTHTGEKPFECKECHKRFTRDHHLKTHMRLHTGEKPYHCTHCERQFVQVANLRRHLRVHTGERPYACELCTSKFSDSNQLKAHMLIHKGEKPFECKKCLGRFRRRHHLMHHKCPKDESNAGKPRRGRRPKPSSADEDALHSALSSTVPSLHQALHHHHHHHHHLHHLRPPHPSHDDAHRPPPPPTQPAAVPGAIANTAPIDSRPTKRERKPRETRRIIKVSIPYAPHVAPPPTGNNARFIESAAASGVAPAAMVPLSNPPEQTEPEDLSMHGGSDNGDRRRPTSAMSASSTATTCLSLHQQPLSGTPSSTLISVLSQPPVPASCASTTQCIVVDDDKDDDVMLTVDEDGDCSEVAIVEEDDDDDQEDEEKDIRRHPQRKKRRVMSRPVRRKGAHAKGMIHPVSHRVKSTIRAGVVGHGGEEEDDDNDDDEDDAMEKEEEGVEGDDEDESEPENGSSPPCGAVGKQGLIHRSRDQVVLPPRVTGRNGQQPKP

>Pt_Krüppel1_XP_015925394.1_zinc finger_37

MKEGSEGDIGCDDVPTSRDADSTVSNGKVNGSMSMVSSQPRRILSDRPPSMGVTALATYEAEKNNASLTSTSIKTEHGNLPMNGTTLANALLGNFLDGSRGSSGSPGNNGESIVGGIHSTSSNLTGKGIKTEGGQNRDKQFMCKICDRAFGYKHVLQNHERTHTGEKPFECKECSKRFTRDHHLKTHMRLHTGEKPYHCSHCDRQFVQVANLRRHLRVHTGERPYACTLCSSRFSDSNQLKAHLLIHEGIKPFHCKKCSGRFRRRHHLVHHKCPRDEANIGRPRRGRRPRAYDQVPTPLPNSLPILSPVLKERLSTPLSDLTPHPPVSLTSVIKRSNSPTSPPIQPPAAHMQAAHMMHLTLGHISNFPLRPNSNGRYYEEVQTGPIDMTVQSSVTSNSVSVIVPRIPQQNNNNYHSSNIHSSLDVVLDLSNSKSDSEAEPIEEEVDEEDDCMDEGLDSESEEDDHSHLRLVSWKDDFHHHPKDLRRRNGDRYDSEGESSNLALQLKLKIN

>Pt-Krüppel2_AHC88001.1

MKDGPEVAEGVKTASSATNGGGDKCDMRSLDEQSRRNGVAEVAVDINALPVGGTLITDAASTNGDSDKTKNGVPKNGEKQFFCRICNRSFGYKHVLQNHERTHTGEKPFECPECHKRFTRDHHLKTHMRLHTGEKPYQCSHCDRHFVQVANLRRHLRVHTGERPYACQMCESRFSDSNQLKAHMLIHRGEKPFQCQECLGRFRRRHHLIHHKCPKDESNLGKSRKGRRPKVYEQIHTSDHEDHEDLDGELLPDISDNEENMVDTNNSSSEAEQPSPPSVHPVET

>Ek_Krüppel1

QEFSALNARYTRMAYSLVPAPELPPVKRHRGNQMGAKLKPRVKEKSFVCSVCNRGFGYKHVLQNHERIHRGEKPFPCPECHKTFRREHQLTTHLRVHTGENPYKCTYCTHEFKQLGNLQRHLRVHTGEKPYVCEWCEAKFAGSSSYKAHINLHTGDKPFKCKYCSQQFRLHKSMQRHKMQVHADLFAPAEEFVILADDEHESKISEKMERSSPSIETKPIISTLIPHRPGERKEYYDENEDKEQKGDR

>Ek_Krüppel2

MALVNNVMSASMLTSSMIGQGMVGSNMVNPSLLSSAMMDASWSESEESCQKRQRGNKIGAKLQPRLKERRFVCEVCNKGFGYKHVLQNHERIHNGEKPFECPNCPKKFSREHHLKNHLRQHTGEEPYKCTFCAGTFKQFGTLQRHLRVHTGEKPYICDLCEAKFADRTGYKSHMHLHTGEKPYTCTICNASFRLDKQLRVHIERMHTTVEEFEDAKPISLELLGVGGDKQNKDKHITILQEDGFNISHQVHWKAQKGEGLNVKLEITRDLNRIGDSDSLDESDDRKDPFQCNECHKKFTRKRSLASHMMKHSKSSAMFKCEYCPRRFANIGHLQLHLPWHTDEKRYLCEVCGATFAYKRGFRSHMNVHMDCCSKKKKKKKSATLRASIHEASATVHSSVVVTRVPLNLRELLLSISQACTLFLAQSFIVNLIVGSCIPVYSNHGDFSL
