# Supplementary material for: Panarthropod tiptop/teashirt and spalt orthologs and their potential role as “trunk”-selector genes
Source: EvoDevo. 2021 Jun 2;12:7. doi: 10.1186/s13227-021-00177-y (PMC8173736; doi:10.1186/s13227-021-00177-y)
Supplement: Supplementary file 5 — Additional file 5: Table S1. Primers. [file 13227_2021_177_MOESM5_ESM.docx]

| Gene NAME | 1^st^ / 2^nd^ PCR | Sequence Forward Primer | Sequence Backward Primer |
| --- | --- | --- | --- |
| *Gm-tsh/tio* | 1^st^ | CCGAGGAGGAAACAGG | CCGAGGAGGAAACAGG |
|  | 2^nd^ | GGTGCTGAGGAAGACC | CCGAGGAGGAAACAGG |
| *Ek-tsh/tio* | 1^st^ | GATGAAGCAGATGGAG | GCAGAAGTTTGGGTTG |
|  | 2^nd^ | GTGACTTGGATGGACC | CACTTCCAAACATGGTG |
| *Pt-tsh/tio* | 1^st^ | AGCGAAGCACTGAAGACGAG | AGATCCTGGGATGTAACGGG |
|  | 2^nd^ | GAGACGGAGTAGCAGAAGTG | GTCTACCTCCACCCTCAGTT |
| *Tc-sal* | 1^st^ | CAGCATCGACAGCAAGTACA | GCATATCTGGCGAGGAAGTA |
|  | 2^nd^ | GGAGGAGACCATTCGGAGAA | CCTCGGGTTTTGATTTTCGG |
| *Gm-sal* | 1^st^ | ATGTCTCGTCGGAAACAGCA | TTTGGTGGTGAAAGCCCTCT |
|  | 2^nd^ | TCACCTTTCCTTGCCTCACT | TCACCTTTCCTTGCCTCACT |
| *Pt-sal1* | 1^st^ | CGCAAGCAAAGAAAGCCCAA | AGCGAAACTGAAGGTGACGA |
|  | 2^nd^ | AGCGAAACTGAAGGTGACGA | CTGTCCGATTGTTTCTGGTC |
| *Pt-sal2* | 1^st^ | GCCTTCTGGATAACGGATCA | TACCACAAAAGCGACACCTG |
|  | 2^nd^ | AAGAGGCAGGTCTAGGAGAT | AAGTAAGCATCCTCACCTCC |
| *Ek-sal* | 1^st^ | CGGTCCTCTCATATAATGCC | TTCCTTGGTGTGGCTTCGAT |
|  | 2^nd^ | CTCCTATACCACTTCCTCCT | ACAGGTTGTATTTCGGGAGC |
