# Supplementary material for: Panarthropod tiptop/teashirt and spalt orthologs and their potential role as “trunk”-selector genes
Source: EvoDevo. 2021 Jun 2;12:7. doi: 10.1186/s13227-021-00177-y (PMC8173736; doi:10.1186/s13227-021-00177-y)
Supplement: Supplementary file 6 — Additional file 6: Table S2. Accession Numbers. [file 13227_2021_177_MOESM6_ESM.docx]

| Gene | Accession No |
| --- | --- |
| *Dm*-Tsh | AAA28983.1 |
| *Dm*-Tio | NP_524733.2 |
| *Tc*-Tio/Tsh | EFA10732.1 |
| *Gm*-Tio/Tsh | c59357_g1 |
| *Pt*-Tio/Tsh | XP_008197624 |
| *Ek*-Tio/Tsh | c209731_g4_i1 |
| *Dm*-ZFH1 | NP_476850.1 |

Tiptop/Teashirt phylogeny

| Gene | Accession No |
| --- | --- |
| *Dm*-Sal | NP_723670.2 |
| *Dm*-Salr | NP_523548.1 |
| *Tc*-Sal | XP_008193710.1 |
| *Gm*-Sal | c58464_g3_i2 |
| *Pt*-Sal1 | XP_015922317.1 |
| *Pt*-Sal2 | XM_016062943.1 |
| *Ek*-Sal | c207818_g1_i1 |
| *Af*-Sal | AJ567454.1 |
| *Dm*-Kr | NP_477467.1 |
| *Tc*-Kr | XP_008194043.1 |
| *Gm*-Kr | CBX48486.1 |
| *Pt*-Kr1 | XP_015925394.1 |
| *Pt*-KR2 | AHC88001.1 |
| *Ek*-Kr1 | SAP35441 |
| *Ek*-Kr1 | SAP35442 |

Spalt phylogeny
